# Synthesis of pincer-type extractants for selective extraction of palladium from PGMs: An improved liquid-liquid extraction approach to current refining processes

**DOI:** 10.1038/s41598-017-09053-z

**Published:** 2017-08-18

**Authors:** Muniyappan Rajiv Gandhi, Manabu Yamada, Kazutoshi Haga, Atsushi Shibayama

**Affiliations:** 1 Graduate School of International Resource Sciences, Akita University, Akita, 010-8502 Japan; 2 Research Center for Engineering Science, Graduate School of Engineering Science, Akita University, Akita, 010-8502 Japan; 3 Department of Applied Chemistry Course, Graduate School of Engineering Science, Akita University, Akita, 010-8502 Japan; 4 Department of Earth Resource Engineering and Environmental Science, Graduate School of International Resource Sciences Akita University, Akita, 010-8502 Japan

## Abstract

SCS pincer ligands **1**–**4** were synthesised, and their ability to extract Pd(II) from HCl and HNO_3_ media was studied. The Pd(II) extraction properties of **1**–**4** were compared with those of commercial extractants (DOS and LIX^®^84-I) in kerosene. **1** and **2** showed superior Pd(II) extractability (*E*% = 99.9) relative to DOS and LIX^®^84-I from 0.1–8.0 M HCl and to DOS from 0.1–8.0 M HNO_3_ and mixed HCl + HNO_3_ media. The Pd(II) extraction rate, acid durability, the most suitable organic/aqueous (O/A) phase ratio, and Pd(II) loading capacity of extractants **1**, **2**, and DOS were evaluated. **1** and **2** exhibited a greater Pd(II) extraction rate and Pd(II) loading capacity than DOS. **1** was very stable in acid media (HCl and HCl + HNO_3_), whereas **2** and DOS deteriorated in HCl + HNO_3_. Selective extraction of Pd(II) by **1** and **2** was achieved from a mixed solution containing Pd, Pt, Rh, rare metals, and base metal ions that simulated the leach liquors of automotive catalysts. The back extraction of Pd(II) and reusability of extractants **1** and **2** were studied. The Pd(II) extraction mechanism of **1**–**4** was investigated using FT-IR, UV-visible, and NMR spectroscopy.

## Introduction

Platinum group metals (PGMs = Pd, Pt, Rh, Ru, Ir, and Os) are used in fields as diverse as automotive and industrial catalysts, jewellery, fuel cells, electronics and electric/hybrid vehicles^1^. PGMs exist at very low levels in the earth’s crust together with other base metals and are available only from South Africa, Russia, Canada and Zimbabwe^1^. The PGM concentration^1^ in primary ores is about 10 g tonne^−1^. A stable supply of PGMs is crucial, because the metals are expensive, and their global market prices fluctuate with demand. The automobile catalyst industry uses *ca*. 40, 58, and 83%, respectively, of the world production of Pt, Pd, and Rh^2^. Automobile catalytic converters contain ~2 g PGMs per converter and have a 10-year lifespan on average. After losing their catalytic properties, the converters become a secondary resource. The PGM content of spent converters (1–2 g kg^−1^) is greater than that of primary ores (~0.01 g kg^−1^)^3, 4^. Recycling of PGMs from secondary resources such as spent automotive and industrial catalysts is economically important^5–9^, reduces the environmental burden of PGM mining, and can limit pollution. High selectivity is required in the separation of PGMs from primary ores and recycling of PGMs from spent automotive catalysts. Hydrometallurgical refining is less costly than pyrometallurgical refining. Current PGMs refineries such as Lonmin (Brakpan, South Africa), Krastsvetment (Kranoyarsk, Russia), Impala Platinum (Springs, South Africa), Vale (Acton, UK), Johnson Matthey (Royston, UK)^10, 11^, Anglo American Platinum (Rustenburg, South Africa), Lonrho (MINTEK, South Africa)^11, 12^, and Heraeus (Hanau, Germany) carry out hydrometallurgical refining by leaching precious metals from concentrated primary and secondary sources using HCl + Cl_2_^1, 11^. The liquors obtained from primary ore leaching contain Au and PGMs as chloro complexes. Secondary sources including automotive catalysts are leached with HCl/Cl_2_, HCl/H_2_O_2_, or aqua regia^2, 5^. The corresponding liquors contain Pd, Pt, and Rh and other rare earth and base metals depending on the manufacturer. The Vale, Johnson Matthey, Anglo American Platinum, Lonrho, Sumitomo Metal Mining (Ehime, Japan)^12^, and Heraeus refineries use solvent extraction to recover PGMs from acid-leached liquors. Other refineries use precipitation and ion-exchange processes^1, 10, 11^. Commercial extractants such as di-*n*-hexyl sulphide (DHS), di-*n*-octyl sulphide (DOS), and 2-hydroxy-5-nonylacetophenone oxime (LIX^®^84-I)^1, 11^ are used in the solvent extraction process. Although these reagents are useful in separating Pd(II) and Pt(IV), they are not without issues regarding extraction rate, selectivity, and durability. The extractants can be oxidised by contact with a highly acidic aqueous phase, which renders Pd(II) separation ineffective. Johnson Matthey and Anglo Platinum refineries recover Pd(II) from acidic leach liquors with LIX^®^84-I. The kinetics of Pd(II) extraction using LIX^®^84-I are extremely slow, and it is necessary to use an organic amine as an accelerator to increase the extraction rate^1^. However, use of an organic amine decreases the selectivity of Pd(II) extraction. LIX^®^84-I extracts Pd(II) from pH 1–3 HCl media with properties that depend greatly on the acidity of the aqueous phase^13, 14^. The raffinate contains < 5 mg L^−1^ Pd(II) after extraction^1^. The Vale, Lonrho and MINTEK refineries use DHS or DOS to separate Pd(II) from acid leach liquors^1, 10–12^. Pd(II) extraction kinetics with DHS or DOS are slow, but < 1 mg L^−1^ Pd is retained in the raffinate after extraction^1^. DHS and DOS are oxidised to di-*n*-hexyl sulphoxide (DHSO) and di-*n*-octyl sulphoxide (DOSO), respectively, during extraction by contact with oxidising agents in the acidic aqueous phase^1, 15–17^. Oxidised DHS and DOS extract Fe(III), Rh(III) and Pd(II) considerably, which decreases the selectively of Pd(II) extraction^1, 15, 16^. Many researchers have employed thiodiglycolamide^18–20^ and acyclic dithioethers^21, 22^ to selectively extract Pd(II) from PGM-containing solutions. Commercial hydrocarbon fluids (kerosene and ISOPAR M) have been used as diluents in PGMs refineries. Many synthesised and commercial extractants^23–36^ exhibit slow extraction rates and extract other PGMs and base metals in minor amounts in addition to Pd ions, which affects the selectivity of extraction. In these cases, extraction removes only Pd(II) into the organic diluent, chlorinated diluent, or mixture of diluents^18–22, 28–33^, and oxidation in acidic media causes Pd(II) extraction to be highly dependent on the acidity and type of leach liquors. Pd(II) extraction efficiency is good in weakly acidic media, but decreases dramatically in strong acid media^22, 31, 33–35^. Synthesis of new, robust extractants faces several major issues including use of expensive reagents, multiple synthetic steps^22, 31^, advanced purification methods, durability, and difficulties in back extraction and reusability. To date, no effective extractants applicable to industrial operations have been developed for complete recovery of Pd(II) from primary and secondary PGM resources. Development of novel, inexpensive extractants possessing a high extraction rate, selective and efficient Pd(II) separation, and superior durability in acidic media is required. Our goal is to develop new extracting agents for the selective and efficient recovery of Pd(II) from leach liquors of automotive catalysts. We previously used the 1,3-bis(dimethylthiocarbamoyloxy)benzene pincer ligand in various organic solvents to selectively extract Pd(II) from the leach liquors of automotive catalysts^30^. Unfortunately, the solubility of 1,3-bis(dimethylthiocarbamoyloxy)benzene in hydrocarbon diluents is low, which limits its industrial application^30^.

The objective of our research is to synthesise new SCS pincer-ligand extractants that are soluble in commercial hydrocarbon diluents and capable of effectively separating Pd(II) in the presence of other PGMs from the acid leach liquors of automotive catalysts. We designed the 1,3-bis(2-(octylthio)propan-2-yl)benzene ligand (**1**) having two *n*-octyl moieties to increase hydrophobicity as a pincer-type extractant to achieve this goal. We also synthesise﻿d the SCS pincer-type extractants 1,3-bis(octylthio)methyl)benzene (**2**), dimethyl[1,3-phenylenebis(1-methylethylidenethio)]diacetate (**3**) and dimethyl[1,3-phenylenebis(methylenethio)]diacetate (**4**) and compared their properties with **1**. Extractant **1** can be synthesise﻿d at the gram to kilogram level in high yield at room temperature from commercially available reagents via a one-step reaction with simple workup procedures. **1** and **2** exhibit large extraction capacities and Pd(II) selectivities compared with **3**, **4**, and commercial LIX^®^84-I and DOS extractants. Extractant **2** exhibits behaviour similar to **1** and is suitable for Pd(II) extraction from HCl media, but is not recommended for HCl + HNO_3_ media. The Pd ion extraction mechanisms have been confirmed using various spectroscopic methods. Pd(II) extractability of extractant **1** is higher than commercial DOS and it can be used for the successful separation Pd(II) in PGM refineries.

## Results and Discussion

### Extraction of Pd(II) from HCl media with 1‒4, DOS, and LIX^®^ 84-I

**Figure 1:**
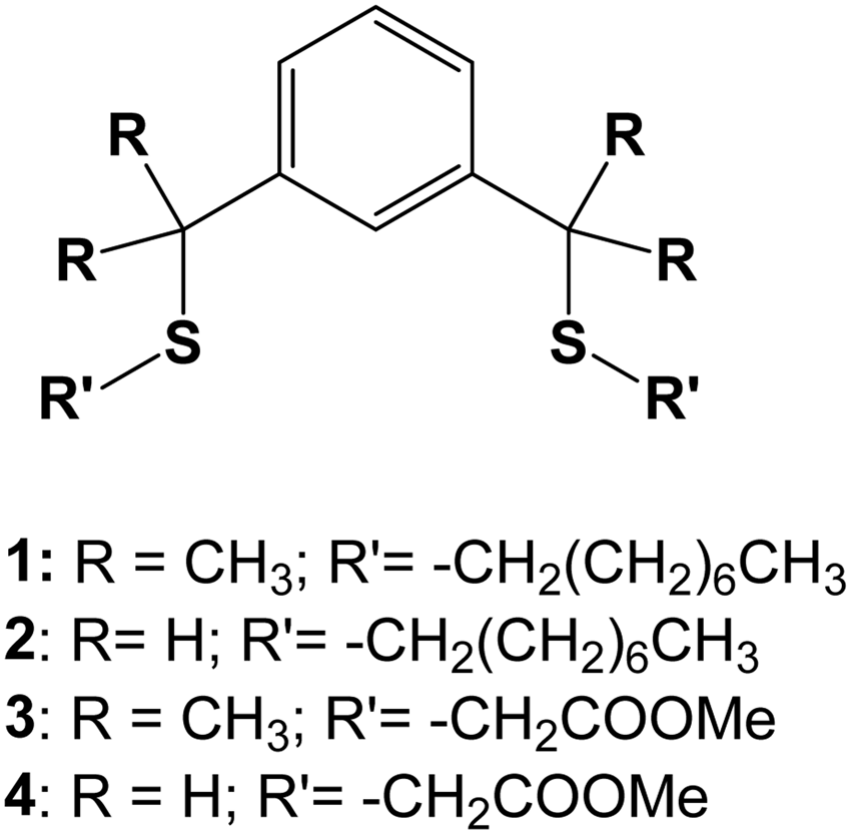
Structures of synthesised SCS type pincer based extractants (**1**‒**4**).

**Figure 2:**
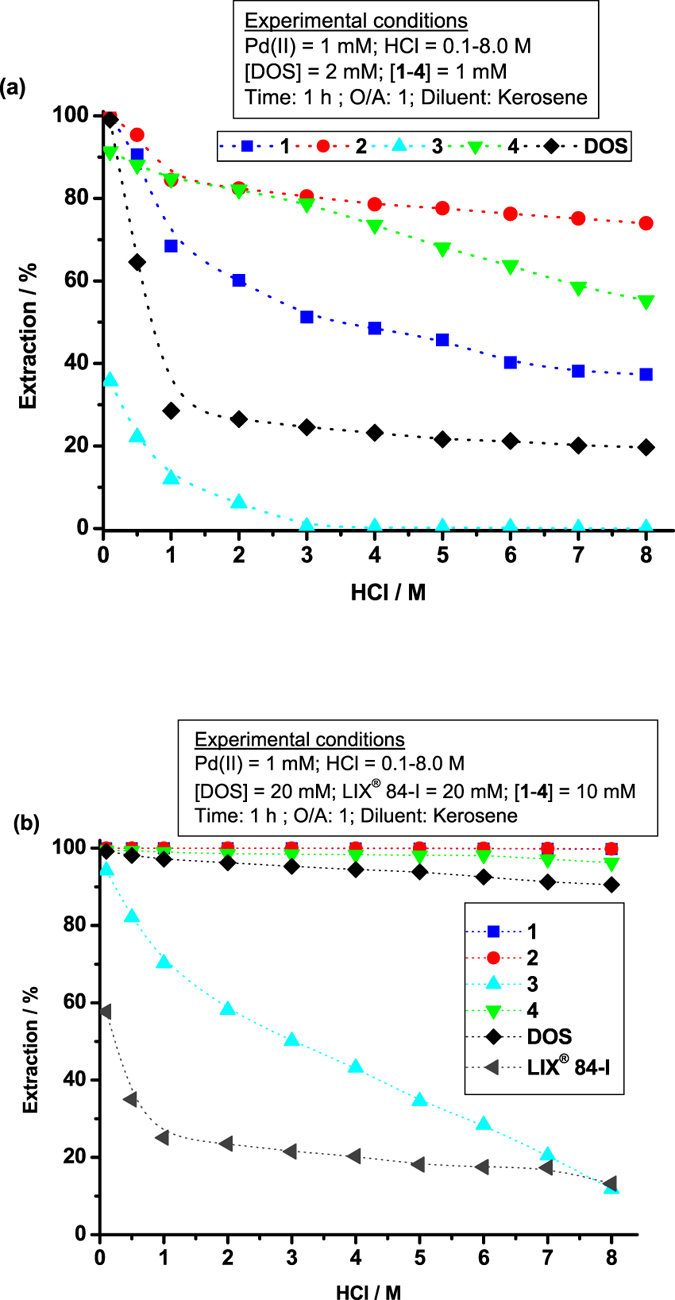
**(a)** The effect of HCl concentration on extraction of Pd(II) by **1**–**4** and DOS. **(b)** Pd(II) extraction ability of extractants **1**–**4**, DOS and LIX^®^ 84-I in HCl media.

The structures of the SCS-type pincer extractants (**1**–**4**) are shown in Fig. 1. Extractants **1**‒**4** contain two sulphur atoms, whereas DOS has only one. To establish sulphur atom equivalence in comparative studies, the concentration of DOS was maintained at twice that of **1**‒**4**. Pd(II) extraction was studied as a function of HCl concentration from 0.1‒8.0 M HCl using 1.0 mM **1**‒**4** and 2.0 mM DOS in kerosene for 60 min. The effect of HCl concentration on Pd(II) extraction is shown in Fig. 2(a). The Pd(II) extraction percentage (*E*%) of **1**, **2**, and DOS was > 99.1% in 0.1 M HCl; extractants **4** and **3** exhibited 91.4 and 35.7% efficiency, respectively. The *E*% of all extractants decreased with increasing HCl concentration. The *E*% of **1**, **2﻿﻿**, and **4** in 1.0 M HCl equalled 68.4, 84.4, and 84.7%, respectively, but that of **3** and DOS was only 11.9 and 28.5%, respectively. In 8.0 M HCl, the Pd(II) extractability of **2**, **4**, **1**, and DOS decreased to 73.9, 55.2, 37.3, and 19.6%, respectively. The Pd(II) extraction capacity of **3** was nil from 3.0‒8.0 M HCl. The experimental results show a distinct dependence of *E*% on HCl concentration and extractant structure.

The extractant concentration was increased ten-fold to increase *E*% in concentrated HCl (*cf*. Fig. 2(a)), and the extraction efficiency of **1**‒**4** was compared to that of commercial LIX^®^ 84-I in addition to DOS. The Pd(II) extractability at 0.1‒8.0 M HCl using 10 mM **1**‒**4** and 20 mM DOS or LIX^®^ 84-I are shown in Fig. 2(b). Photographs of the Pd(II) extraction behaviour of **1**‒**4** in kerosene are shown in supplementary Fig. S1. The experimental conditions are those referred in Fig. 2 with 0.1 M HCl solution. Extractant **4** produced 99.4‒96.2% and extractants **1** and **2** > 99.7% Pd(II) extraction from 0.1‒8.0 M HCl. Ligand **4** formed a third phase (emulsion) during extraction, and the resulting Pd(II) complex was not soluble in kerosene as shown in the photograph in Fig. S1. Although the > 96.2% extraction efficiency of **4** is very high in all HCl media, it cannot be used in industrial applications because of third phase formation. The *E*% of **3** was 94.2% in 0.1 M HCl, 11.8% in 8.0 M HCl, and decreased steadily with increasing HCl concentration. The *E*% of DOS equalled 99.1–97.1% in 0.1−1.0 M HCl and decreased to 90.5% in 8.0 M HCl. LIX^®^ 84-I exhibited an *E*% of only 57.7–35.0% at 0.1 M–1.0 M HCl^13, 14^ and was 13.2% in 8.0 M HCl. Among all extractants, **1** and **2** at 99.9–99.7% and DOS at 99.1–90.5% exhibited the highest Pd(II) extractabilities in HCl. Evident phase separation was observed for **1**‒**3**, DOS and LIX^®^ 84-I during extraction. Because **3** and LIX^®^ 84-I exhibited poor efficiency in concentrated HCl and **4** formed a third phase, further studies were limited to **1**, **2**, and DOS.

### Pd(II) extraction rate of 1, 2, and DOS in HCl media

**Figure 3:**
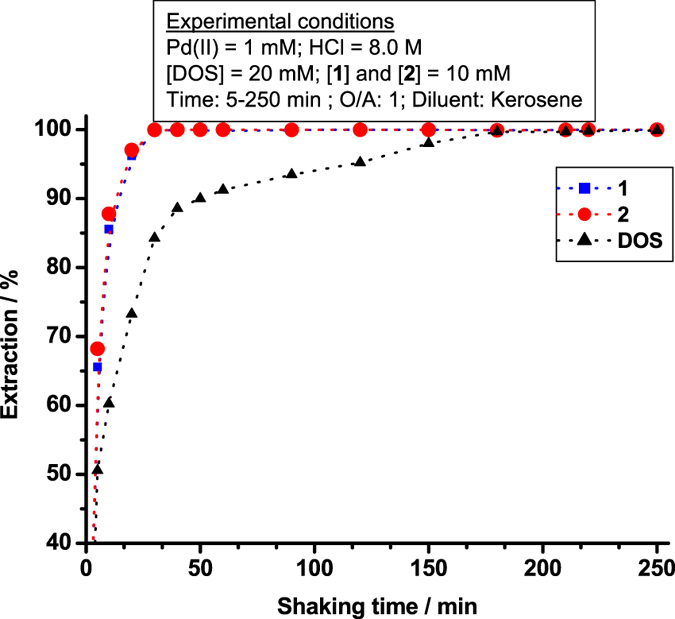
Pd(II) extraction rate of **1**, **2** and DOS in HCl media.

The Pd(II) extraction rates of **1**, **2**, and DOS in 8.0 M HCl were determined by varying the contact time from 5–250 min. Results are shown in Fig. 3.

Extraction by **1** and **2** reached saturation within 30‒60 min, whereas that by DOS required 180 min^1, 19^. Many researchers have reported that slow Pd(II) extraction kinetics by DOS is a major disadvantage in PGM refineries^1, 11^. The superior extractability and extraction kinetics of **1** and **2** relative to DOS may derive from Pd(II) coordination by adjacent sulphur atoms in the pincer ligand. Thus, the SCS pincer-type reagents **1** and **2** are more potent Pd(II) extractants than DOS. The Pd(II) extraction mechanism of **1** and **2**, which differs from that of DOS will be discussed in later section. We previously reported the rapid and selective extraction of Pd(II) using the SCS pincer ligand 1,3-bis(dimethylthiocarbamoyloxy)benzene and described the extraction mechanism in terms of intramolecular coordination^30^.

### Extraction of Pd(II) from HNO_3_ and mixed HCl-HNO_3_ media

**Figure 4:**
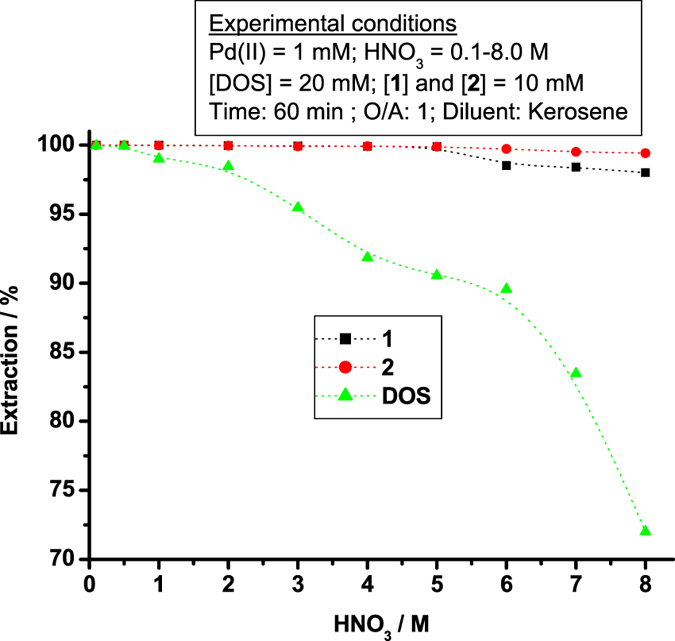
Extraction of Pd(II) from HNO_3_ media using **1**, **2**, and DOS.

In many cases, PGM-leached liquors are prepared from spent automotive and industrial catalysts using HNO_3_ and aqua regia^37–39^. To check the Pd(II) extraction ability of **1**, **2**, and DOS in HNO_3_ or mixed HCl + HNO_3_ solution, studies were conducted using 10 mM **1** or **2** or 20 mM DOS with 1 mM Pd(II) from 0.1 M–8.0 M HNO_3_ and mixtures of 0.1 M HCl + 0.25 M HNO_3_, 1.0 M HCl + 0.50 M HNO_3_, 2.0 M HCl + 0.75 M HNO_3_, 3.0 M HCl + 1.0 M HNO_3_, and 6.0 M HCl + 2.0 M HNO_3_. Pd(II) extraction from HNO_3_ media are shown in Fig. 4.

Ligands **1** and **2** showed 99.9‒98.0% Pd(II) extraction from 0.1‒8.0 M HNO_3_. DOS showed 99.9-99.0% Pd(II) extraction from 0.1‒1.0 M HNO_3_ with *E*% decreasing with increasing HNO_3_ concentration to a low of 72.0% at 8.0 M HNO_3_. Thus, pincer ligands **1** and **2** exhibit nearly quantitative extraction of Pd(II) in nitric acid media and can be used on a par with DOS in refineries. Extractant capability also was studied in mixed HCl‒HNO_3_ media. Pd(II) extraction was performed using 10 mM **1** or **2** or 20 mM DOS in kerosene with 1 mM of Pd(II) from mixtures of 0.1 M HCl + 0.25 M HNO_3_, 1.0 M HCl + 0.50 M HNO_3_, 2.0 M HCl + 0.75 M HNO_3_, 3.0 M HCl + 1.0 M HNO_3_, and 6.0 M HCl + 2.0 M HNO_3_. The Pd(II) *E*% values in mixed HCl‒HNO_3_ media using **1**, **2**, and DOS are shown in Fig. S2. The *E*% of **1** and **2** was > 99.9% in all cases. The *E*% of DOS was 96.2% in 0.1 M HCl + 0.25 M HNO_3_, 85.2% in 1.0 M HCl + 0.50 M HNO_3_, 74.5% in 2.0 M HCl + 0.75 M HNO_3_, 66.4% in 3.0 M HCl + 1.0 HNO_3_, and 48.5% in 6.0 M HCl + 2.0 M HNO_3_. Although the Pd(II) extractability of DOS varies dramatically with composition in mixed HCl + HNO_3_ mixed media, **1** and **2** can be used in acid-leached liquors containing Pd(II) without regard to the HCl and/or HNO_3_ content.

### Durability of 1, 2, and DOS in various acid solutions

Commercial DOS and DHS^40^ and other reagents have been reported to deteriorate upon repeated extraction of PGMs from leach liquors^1, 15, 19^. Extractant degradation has been attributed to oxidation or other reactions upon contact with acid-leached solutions, which decreases extraction capacity and reusability. Deteriorated reagents also co-extract undesired metals ions^15^. To study the acid durability of **1**, **2**, and DOS, 1 mL of each extractant was diluted in 10 mL of CHCl_3_ and stirred with 10 mL of 2.0 M HCl + 1.0 M HNO_3_ or 12.0 M HCl at room temperature (20 ± 1 °C) for 7 days. The CHCl_3_ phase was separated and evaporated, and each acid-treated extractant was characterised by FT-IR and^1^H NMR spectroscopy.

**Figure 5:**
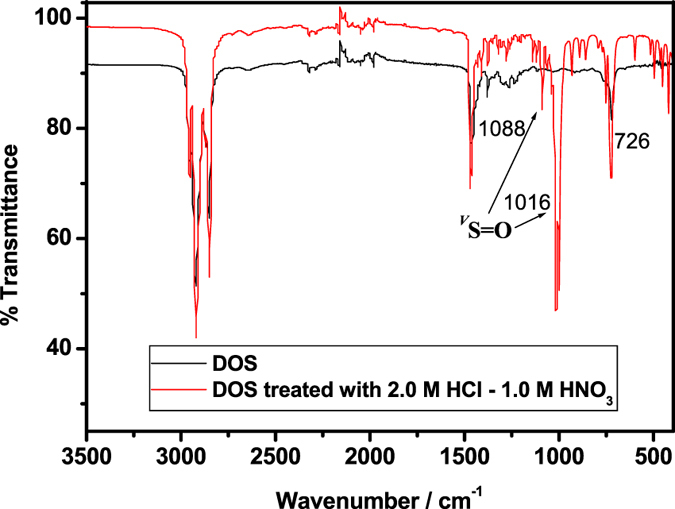
FT-IR spectra of native DOS and 2.0 M HCl + 1.0 M HNO_3_ treated DOS.

FT-IR spectra of native DOS and DOS treated with 2.0 M HCl + 1.0 M HNO_3_ are shown in Fig. 5.

The FT-IR spectra of acid-treated DOS showed new sharp peaks at 1016 and 1088 cm^−1^ corresponding to protonated –S=O and free –S=O, respectively^19^. The HCl + HNO_3_ medium oxidises the sulphide group of DOS to sulphoxide^19, 41^, consistent with reports that DHS and DOS are prone to oxidation upon acid contact^1, 15, 19, 41^. The ^1^HNMR spectra of native and acid-treated DOS are shown in Fig. S3. The ^1^H NMR spectrum of acid-treated DOS differs from that of native DOS due to oxidation, and corresponds exactly to the standard ^1^H NMR spectrum of di-*n*-octyl-sulphoxide^42^. The FT-IR and ^1^H NMR spectra clearly show the deterioration of DOS by transformation into DOSO upon acid contact. The FT-IR spectra of native **2** and **2** treated with 2.0 M HCl + 1.0 M HNO_3_ are illustrated in Fig. S4. Acid-treated **2** differs from native **2** by showing new peaks at 1051 and 1088 cm^−1^, which correspond to –S=O^42^. The ^1^HNMR spectrum of **2** (Fig. S5) also changed significantly upon 2.0 M HCl + 1.0 M HNO_3_ treatment confirming its oxidation in a manner similar to that of DOS^42^.

The FT-IR and ^1^HNMR spectra of **1** treated with 2.0 M HCl + 1.0 M HNO_3_ are shown in Figs. S6 and S7. In these spectra, the acid-treated extractant shows peaks corresponding exactly to its native form. This result confirms that extractant **1** is stable in 2.0 M HCl + 1.0 M HNO_3_ and that its sulphur atoms are not oxidised by acid treatment. The acid resistance of the sulphur atoms in **1** may be due to the steric hindrance of the neighbouring methyl groups, which would impede direct approach of acidic components to the sulphur atoms.

The FT-IR and ^1^H NMR spectra of extractants **1**, **2**, and DOS treated with 12.0 M HCl did not change and exactly matched the spectra of their native forms. Thus, **1**, **2**, and DOS are not oxidised or otherwise degraded in HCl. Extractant **1** was very stable and displayed high oxidation resistance in HCl and HNO_3_, whereas DOS and **2** were stable only in HCl. The oxidation of DHS, DOS, and other sulphur-based extractants in acid media decreases the efficiency and selectivity of Pd(II) extraction^1, 15, 19, 21^.

**Figure 6:**
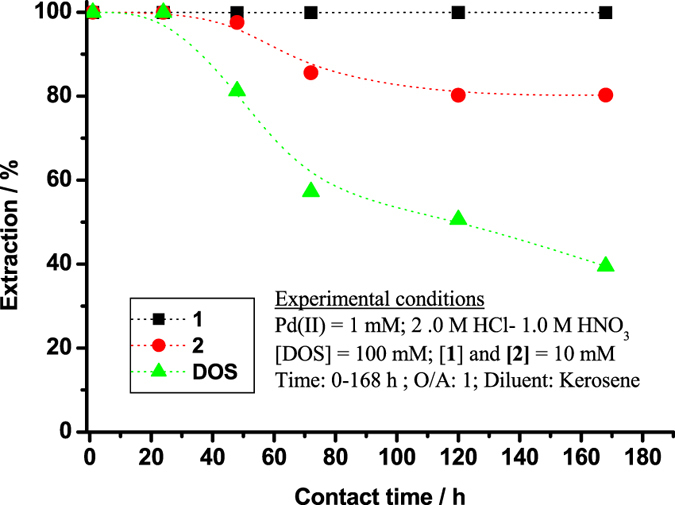
Pd(II) extractability of **1**, **2** and DOS in 2.0 M HCl + 1.0 M HNO_3_ media at various time intervals.

PGMs generally are leached from primary and secondary resources using HCl + Cl_2_ or aqua regia^1, 36–39^. The acid leach liquors contain Cl^−^ ions and have the potential to oxidise extractants over time. To examine the effect of such deterioration on **1**, **2**, and DOS, Pd(II) extractability from HCl + HNO_3_ was measured at various intervals. Results are presented in Fig. 6. The Pd(II) *E*% of **1** was 99.9% from 1 h to 7 days. On the other hand, the *E*% of DOS and **2** equalled 99.9% from 1 h to 24 h, whereafter efficiency declined gradually with increasing contact time to 80.2% for **2** and 39.5% for DOS after 7 days. The oxidised product of DOS (DOSO) is sparingly soluble in kerosene, which may adversely affect extraction efficiency.

Okuda *et al*.^15^ studied the extraction of Pd(II) and Pt(IV) from HCl and HNO_3_ and reported the degradation of DHS to DHSO. Degradation of DHS in HCl increased slowly upon repeated operations, but DHS was oxidised immediately in HNO_3_. Presence of DHSO and DHS in the organic phase also induced formation of an insoluble third phase between the organic and aqueous phases in mixed-metal solution extractions. Sato *et al*.^41^ reported that the Pd(II) extraction efficiency of DHS was greater than that of DHSO in HCl. Pd(II) is a soft acceptor and tends to bind more strongly to soft donor atoms. Oxidation of the “soft” sulphur donor(s) in DOS and **2** to “hard” sulphoxide group donor(s) in the degraded products inhibits Pd(II) extraction. A further disadvantage of sulphoxide and protonated sulphoxide groups in oxidised extractants is the uptake of undesired metal ions. Among the compounds studied, **1** is highly resistant to oxidation in acidic media and is suitable for long-term use in industrial applications.

### Determination of the best organic/aqueous (O/A) phase ratio and loading capacity

The optimum O/A phase ratio for Pd(II) extraction was determined by varying the phase ratio from 1:5 to 1:1 using 10 mM **1** or **2** or 20 mM DOS with 1 mM Pd(II) in 8.0 M HCl. The effect of the O/A ratio on Pd(II) extraction is shown in Fig. S8. The *E*% of extractant **1** ranged from 99.9 to 99.0% at 1:1 to 1:3 O/A. The efficiency was 95.3 and 90.8% at O/A = 1:4 and 1:5, respectively. The efficiency of extractant **2** was > 99.5% from 1:1 to 1:5 O/A. The *E*% of DOS varied from 92.1 to 85.9% at O/A = 1:1 to 1:5. These results confirm that **1** and **2** extract Pd(II) to > 99.9% and leave < 0.05 mg L^−1^ in the aqueous phase at a 1:1 O/A ratio, whereas DOS has a maximum 92.1% extraction efficiency and leaves ~ 8.4 mg L^−1^ Pd(II) in the aqueous phase at 1:1 O/A. The optimum O/A for complete extraction of Pd(II) with **1** and **2** is 1:1.

The effect of Pd(II) loading was studied using 10 mM **1**, **2**, or DOS with 1 mM Pd(II) in 8.0 M HCl. The Pd(II) loading capacities are illustrated in Fig. S9. With 10 mM extractants in the organic phase, the loading capacities contrast the 1:1 Pd/extractant ratio for **1** and **2** to the 1:2 Pd/extractant ratio for DOS^40, 41^ upon saturation with 1.2 g L^−1^ Pd(II) at 8.0 M HCl. The loading capacities were ~ 1.06 g L^−1^ (10 mM) for **1** and **2** and ~ 0.53 g L^−1^ (5 mM) for DOS. Because the SCS pincer ligands bind twice as much Pd(II) as DOS, two moles of DOS are required to extract one mole of Pd(II) in HCl media^40, 41^, whereas a single mole of **1** or **2** is sufficient to extract one mole of Pd(II). Thus, **1** and **2** are more economical than DOS or DHS for Pd(II) extraction.

### Effect of diluents on the extraction of Pd(II) ions by 1 and 2

Ligands **1** and **2** provide excellent Pd(II) extraction and good phase separation in kerosene. However, many PGM refineries use odourless and safe hydrocarbons such as ISOPAR M and ShellSol D70^®^ that possess a high flash point (similar to kerosene) and reduce the risk of environmental exposure. We therefore evaluated Pd(II) extraction by **1** and **2** using the aliphatic hydrocarbon diluents ISOPAR M, ShellSol D70^®^, *n*-decane, *n*-dodecane, and kerosene and aromatic diluents such as *p*-xylene and toluene. The effect of these diluents on the extraction by **1** and **2** are given in Table 1. The efficiency of extraction with aliphatic diluents was 99.7‒99.9% in kerosene, ISOPAR M, ShellSol D70^®^, *n*-dodecane, and *n*-decane. *E*% was 97.8–98.7% with *p*-xylene and toluene. Pd(II) extraction efficiency was excellent in all cases indicating **1** and **2** can be used with most diluents in industrial applications.

**Table 1:** Effect of diluents on the extraction of Pd(II) by 1 and 2.

Diluents	Boiling point (°C)	Flash point (°C)	Extractability %	
			**1**	**2**
Kerosene	150–300	38–66	99.9	99.9
ISOPAR M	218–257	96	99.9	99.9
ShellSol D70^®^	190–250	78	99.9	99.9
*n*-Dodecane	216	71	99.9	99.9
*n*-Decane	174	46	99.7	99.8
*p*-Xylene	138	25	97.8	98.1
Toluene	110	4.4	98.1	98.7

Conditions: Pd(II) = 1 mM in 3.0 M HCl; [E] = 10 mM; Time = 60 min; O/A = 1;

Shaking speed = 300 rpm.

### Extraction of Pd(II) from simulated mixtures of PGMs, rare metals, and base metal ions

**Figure 7:**
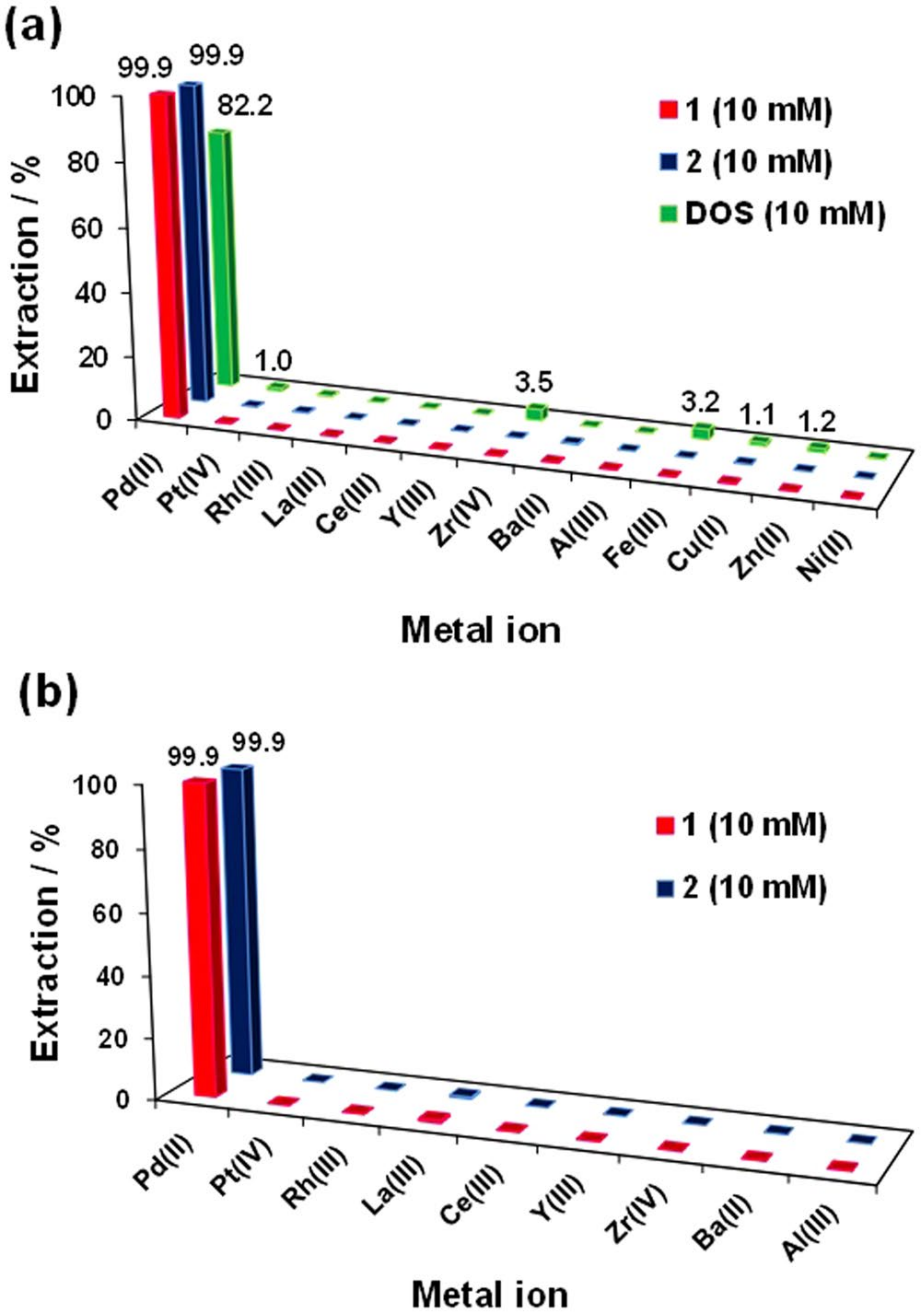
**(a)** Extraction of Pd(II) from simulated mixed solution containing PGMs, rare metals, and base metal ions by **1**, **2**, and DOS. **(b)** Pd(II) extractability of **1** and **2** from leach liquors of automotive catalysts.

PGM-leached liquors from automotive catalysts typically contain various PGMs (Pt, Pd and Rh), rare metals (Zr, Y, La and Ce), alkaline earth metals (Ba), and base metals (Cu, Fe, Zn, and Ni)^21, 43^. New extractants must be able to extract Pd(II) selectively from these mixtures. An extraction experiment was conducted with a simulated mixed-metal solution containing 0.1 g L^−1^ each of Pd, Pt, Rh, Y, Zr, Ba, Al, La, Ce, Cu, Fe, Zn, and Ni in 3 M HCl using 10 mM **1** or **2** or 20 mM DOS. Results are presented in Fig. 7(a). Extractants **1** and **2** removed 99.9% of the Pd(II) from the simulated solution, which demonstrates their obvious selectivity for this metal. Extraction of other metals was negligible. The *E*% of DOS was 82.2%, and minor amounts of Zr(IV), Fe(III), and other base metals were co-extracted^15^. Extractants **1** and **2** are therefore very suitable for extracting Pd(II) from leach liquors containing various platinum group, rare earth, and base metals.

### Pd(II) recovery from leach liquors of automotive catalysts

Automotive catalysts are important secondary sources of PGMs. The catalysts typically contain 1‒2% PGMs and 90% supporting materials such as Al_2_O_3_, La_2_O_3_, CeO_2_, ZrO_2_, BaO, and other metal oxides. Our group has reported the leaching of automotive catalysts using HCl (11.6 M) + H_2_O_2_ (1 vol%)^43^. To study the leaching process, we prepared liquors of automotive catalysts containing Pd, Pt, Rh, Zr, Ce, Ba, Al, La, and Y. The metals exist as chloro complexes in concentrated HCl solution, which was diluted five- fold with water before Pd(II) extraction studies. The metal concentrations are given in Table 2. The extraction of Pd(II) was conducted by shaking 10 mL of the diluted leach liquors and 10 mL of kerosene containing 10 mM **1** or **2** for 60 min. The extraction results are illustrated in Fig. 7(b). Extractants **1** and **2** displayed selective and nearly quantitative removal of Pd(II) (*E*% = 99.9%). No other metals were extracted from the leach liquors by **1** and **2**. Because of the ability of the pincer-like structure to capture metal ions via soft-base sulphur atoms, extractants **1** and **2** can selectively extract Pd(II) from leach liquors by intramolecular coordination^30^.

**Table 2:** The metal concentrations in the diluted leach liquors of the automotive catalysts.

Metal ions	[M]_aq,init_ (mg/L)
Pd(II)	92.2
Pt(IV)	53.5
Rh(III)	36.8
La(III)	87.3
Ce(III)	606.9
Y(III)	3.5
Zr(IV)	24.6
Ba(II)	287.3
Al(III)	318.7

The Pd(II) loading capacity of **1**, **2**, or DOS (10 mM) from leach liquors of automotive catalysts with O/A = 1 for 60 min were carried out and results are given in Fig. S10. The metal concentrations in the leach liquors are those referred in Table 2. The capacity of **1** and **2** was found to be ~1.057 g L^−1^ from the leach liquors. On the other hand, DOS loaded ~ 0.528 g L^−1^ of Pd(II). In fact, the Pd(II) loading capacity of **1** and **2** is 2**-**fold higher than that of DOS (*cf*. Fig. S9).

### Back extraction of Pd(II) and reusability of extractants 1 and 2

**Figure 8:**
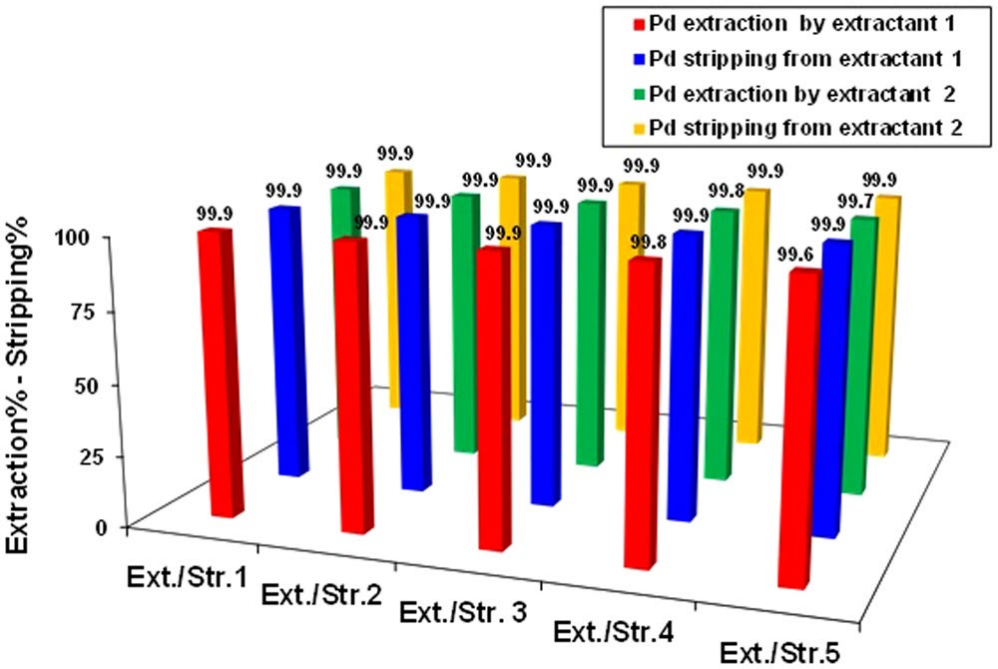
Pd(II) extraction and stripping and their reusability of **1** and **2**.

A successful process includes efficient back extraction to facilitate the reuse of extractants in subsequent operations. Following selective extraction of Pd(II) from the leach liquors of automotive catalysts with **1** and **2**, back extraction of Pd(II) from the organic phase was carried out using five stripping agents: 1 M HCl, 1 M HNO_3_, 1 M H_2_SO_4_, 5% aqueous NH_3_, and a mixture of 0.1 M thiourea and 1 M HCl. Stripping was conducted with equal volumes of the Pd(II)-loaded organic phase and stripping solution for 60 min. The 1 M HCl, 1 M HNO_3_, 1 M H_2_SO_4_, and 5% aqueous NH_3_ stripping solutions performed poorly due to strong coordination between the extractants and Pd(II). Our group^30^ and other researchers^20, 21^ have reported acidic thiourea to be a powerful stripping agent for back extraction of Pd(II). A solution containing 0.1 M thiourea and 1 M HCl was used to achieve the optimum stripping percentage (*S*%) of Pd(II). Complete back extraction of Pd(II) from organic phases of **1** and **2** by thiourea + HCl was confirmed by *S*% values greater than 99.9%. After back extraction, 10 mL portions of the organic phase were washed with 20 mL of water and reused over four additional cycles of extraction and back extraction under the same experimental conditions. The efficiency of recycled **1** and **2** was > 99.6% after five extraction/stripping cycles. This reusability is demonstrated in Fig. 8. The colour changes of the kerosene phases of **1** and **2** during stripping experiments are shown in Fig. S11.

### Pd(II) extraction mechanism of extractants 1–4

The extraction mechanism of ligands **1**–**4** was studied using the corresponding extractant-Pd complexes. The complexes were prepared by extraction of 1.0 mM Pd(II) from HCl with 1.0 mM **1**–**4** in CHCl_3_. After extraction, the aqueous and organic phases were separated, and the CHCl_3_ phase was evaporated to dryness to obtain the extractant-Pd complex. The FT-IR spectra of **1** and **2** and their Pd complexes are shown in Fig. S12. The FT-IR spectra of **1** and **2** change significantly upon complexation of Pd(II). The C−S peak of **1** and **2** shifts from 703 to 727 cm^−1^ and from 708 to 720 cm^−1^, respectively. The FT-IR spectra of **3** and **4** show similar changes after Pd(II) extraction (Fig. S13). The shifts of the C−S and C−H peaks to higher frequency and the appearance of new peaks suggest that Pd(II) extraction occurs by coordination to the sulphur atoms. The mechanism of Pd(II) extraction by **1**–**4** also was studied using UV-visible spectroscopy. UV-visible spectra of **1**–**4**, an aqueous Pd(II) solution, and the extractant-Pd complexes are shown in Fig. S14. Solutions of **1**–**4** in CHCl_3_ exhibit no peaks in the UV-visible region from 250–600 nm. An aqueous Pd(II) solution in 0.1 M HCl shows a strong absorption^44^ at 250–290 nm and a weak absorption at 450 nm, which corresponds to PdCl_4_^2-^. The **1**-Pd and **3**-Pd complexes show a strong broad peak at 280–330 nm and weak broad peaks at 340–400 nm, which correspond to ligand-to-metal charge-transfer (LMCT) transitions^30^. The **2**-Pd and **4**-Pd complexes have a strong LMCT peak at 315 nm. All extractants show new peaks after reaction with Pd(II) confirming Pd-extractant coordination. The UV-visible features of the **1**-Pd and **3**-Pd complexes are similar as are those of the **2**-Pd and **4**-Pd complexes. The colours of the **1**-Pd, **2**-Pd, **3**-Pd, and **4**-Pd complexes differ following extraction. Ligand **1** possesses four methyl groups and produces a yellow-coloured product upon extraction. In contrast, extractant **2**, which possesses no methyl groups, yields an orange product in the organic phase. The colour difference between **1** and **2** after Pd(II) extraction suggests a difference in extraction mechanism. A similar difference in colour upon extraction by **3** (with methyl groups) and **4** (without methyl groups) suggests a similar mechanistic difference.

The difference in Pd(II) extraction mechanism among the SCS pincer-type extractants was confirmed by ^1^HNMR. Carbon numbering in the aromatic ring for extractant **1**‒**4** was given in the Fig. S15. The ^1^H NMR spectra of **1** and **1**-Pd are shown in Fig. S16. After Pd(II) extraction, the aromatic C(2) proton peak is absent, and the integrated aromatic proton intensity is reduced by 1 H^30^. The C(4,6) proton peak shifts from 7.33 to 6.74 ppm, and the C(5) proton peak shifts from 7.26 to 7.03 ppm. The other proton peaks also shift. The results of the ^1^HNMR analysis of **1** and **1**-Pd are collected in Table S1. The ^1^H NMR of **1**-Pd shows that Pd ion is directly bonded to C(2) and that the C(2) hydrogen is removed by Pd complexation. Pd ion therefore bonds to the aromatic C(2) carbon and sulphur atoms of **1** to form a Pd-pincer complex upon extraction^30^. Palladation involving the benzene ring and sulphur moieties is promoted by formation of two fused five-membered chelate rings.

The ^1^H NMR spectra of **2** and **2**-Pd are shown in Fig. S17. The ^1^H-NMR spectrum of **2**-Pd contains the aromatic proton peaks corresponding to **2** ^45^, which shift upon complexation by Pd(II). The results of the ^1^HNMR analysis of **2** and **2**-Pd are shown in Table S2. The ^1^H NMR of **2**-Pd indicates that Pd is not directly bonded to the C(2) carbon upon complexation and does not form an SCS Pd-pincer complex.

**Figure 9:**
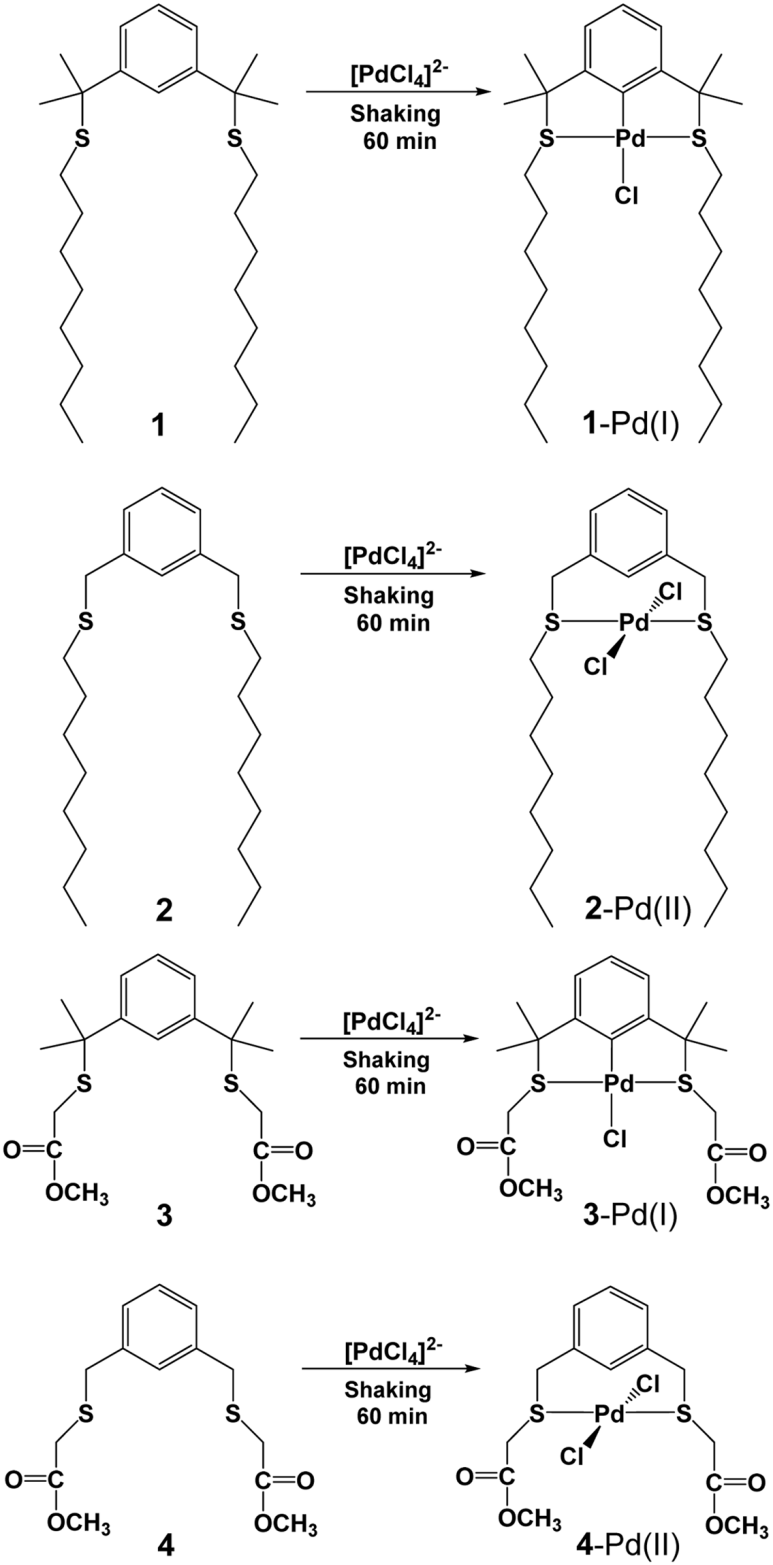
Schematics of feasible mechanism of Pd(II) extraction by **1**‒**4**.

The ^1^H NMR spectra of **3** and **3**-Pd^46^ are shown in Fig. S18. After Pd(II) extraction, the aromatic C(2) proton peak is absent, and the aromatic proton intensity is reduced by 1 H. The C(4,6) proton peak shifts from 7.39 to 6.79 ppm, the C(5) proton peak shifts from 7.28 to 7.09 ppm, and the other proton peaks also shift significantly. The results of the ^1^HNMR analysis of **3** and **3**-Pd are presented in Table S3. The ^1^H NMR of **3**-Pd indicates that Pd is bonded directly to C(2) and that the C(2) proton is removed upon extraction^46^. The ^1^H NMR spectra of **4** and **4**-Pd are shown in Fig. S19. The NMR spectrum of **4**-Pd shows all aromatic proton peaks corresponding to **4** and shifts in all proton peaks upon Pd(II) complexation. The ^1^H NMR results of **4** and **4**-Pd are collected in Table S4. The ^1^H NMR of **4**-Pd indicates that Pd is not bonded to C(2) upon complexation and does not form an SCS Pd-pincer complex^46^. In extractants **1** and **3** ^46^, the steric hindrance generated by the four methyl groups at the benzylic positions facilitates aromatic C(sp^2^)-H bond activation and promotes Pd-C(2) bond formation. Pd complexation at the C(2) carbon of the benzene ring and the sulphur moieties occurs via formation of stable five-membered chelate rings. Yamashina *et al*.^46^. reported the same SCS pincer complex upon reaction of **3** (C_18_H_26_O_4_S_2_) with [PdCl_2_(MeCN)_2_] in CHCl_3_ at room temperature. They reported the crystal structure of the **3**-Pd complex (C_18_H_25_ClO_4_PdS_2_), which showed palladium complexation to the C(2) carbon and sulphur atoms to form five-membered chelate rings. Extractant **4** did not form an SCS pincer-type complex, but rather a conventional **4**-Pd complex (C_14_H_18_Cl_2_O_4_PdS_2_), upon reaction of **4** (C_14_H_18_O_4_S_2_) with [PdCl_2_(MeCN)_2_] in MeCN at room temperature. We have observed the same pattern of complexation in this work during liquid–liquid extraction of Pd(II) with **1**–**4**. Based on the ^1^H NMR studies and literature reports, it is concluded that extractants **1** and **3** remove Pd(II) at room temperature by forming a pincer complex via activation of an aromatic C(sp^2^)-H bond. Extractants **2** and **4** are not capable of activating an aromatic C(sp^2^)-H bond at room temperature and do not form a pincer complex. Schematic representations of the Pd(II) extraction mechanisms involving **1**–**4** are presented in Fig. 9.

## Methods

### Materials and methods

Stock solutions of metal ions were prepared using PdCl_2_, NiCl_2_·6H_2_O, ZnCl_2_ (Kanto Chemical Co., Inc.), PtCl_4_ (Acros Organics), RhCl_3_·3H_2_O, FeCl_3_, CuCl_2_·2H_2_O, AlCl_4_, YCl_3_·6H_2_O, ZrCl_4_, LaCl_3_·7H_2_O, BaCl_2_·2H_2_O (Wako Pure Chemical Industries, Ltd.), and CeCl_3_·7H_2_O (Nacalai Tesque, Inc.) in acid solution. α,α’-Dihydroxy-1,3-diisopropylbenzene and 1-octanethiol were purchased from Tokyo Chemical Industry Co., Ltd. ZnI_2_ was obtained from Kanto Chemical Co., Inc. The commercial extractants, di-*n*-octyl sulphide and LIX^®^ 84-I (2-hydroxy-5-nonylacetophenone oxime), were purchased from Tokyo Chemical Industry Co., Ltd. and Cognis Japan Ltd., respectively. The commercial diluents kerosene (Nacalai Tesque, Inc., Japan; boiling point 150–300 °C), ShellSol D70^®^ (Kremer Pigmente, GmbH & Co. KG; boiling point 198–242 °C, 0.2% aromatic content), and ISOPAR M (Tonen General Petroleum Co., Ltd., Tokyo, boiling point 218–257 °C, < 1–2% aromatic content) were used as received. All other diluents and chemicals were obtained from commercial sources and used without further purification. Electronic absorption spectra were recorded on a Shimadzu UV 3600 double-beam spectrophotometer using 1-cm matched quartz cells. Fourier transform infrared (FT-IR) spectra of the extractants, the extractant-Pd complex and the acid treated extractants were measured using a Thermo Fisher Scientific Nicolet iS50 attenuated total reflection (ATR) spectrophotometer. Proton nuclear magnetic resonance (^1^H NMR) data of the extractants, the extractant-Pd complex and the acid treated extractants in CDCl_3_ were recorded on Bruker DPX 300 and JEOL 600SSS ECA-600 instruments at 20 ± 1 °C. Chemical shifts are quoted as parts per million (ppm) relative to the peak of tetramethylsilane (TMS).

### Synthesis of extractants

1,3-Bis(octylthio)methyl)benzene (**2**), dimethyl[1,3-phenylenebis(1-methylethylidenethio)]diacetate (**3**), and dimethyl[1,3-phenylenebis(methylenethio)]diacetate (**4**) were synthesise﻿d according to literature methods^45, 46^. 1,3-Bis(2-(octylthio)propan-2-yl)benzene (**1**) was synthesise﻿d as follows: α,α′-dihydroxy-1,3-diisopropylbenzene (1.55 g, 7.98 mmol), ZnI_2_ (2.56 g, 8.02 mmol), and 1-octanethiol (2.45 g, 16.78 mmol) were stirred in dry 1,2-dichloroethane (50 mL) for 2 h at room temperature (20 ± 1 °C) under a nitrogen atmosphere. The synthetic scheme of extractant **1** is shown in Fig. S20. After reaction, the mixture was diluted with dichloromethane and washed with water. The organic phase was washed with 1 M NaOH and dried over anhydrous Na_2_SO_4_. The solvent was evaporated to obtain **1** as a colourless oil (yield: 3.45 g, 96%). FT-IR (ATR) ν/cm^−1^: 703 (C-S), 1460 (CH_3_), and 2922 (C-H).^1^H NMR (300 MHz, CDCl_3_, TMS) δ 7.76 (t, 1 H, Ar-2-*H*), 7.33 (dd, 2 H, Ar-4,6-*H*), 7.26 (t, 1 H, Ar-5-*H*), 2.18 (t, 4 H, -S-C*H*_2_-CH_2_-(CH_2_)_5_-CH_3_), 1.70 (s, 12 H, -Ar-(C*H*_3_)_2_-S), 1.34 (m, 4 H, −S-CH_2_-C*H*_2_-(CH_2_)_5_-CH_3_), 1.30-1.10 (m, 20 H, -S-CH_2_-CH_2_-(C*H*_2_)_5_-CH_3_), 0.85 (t, 6 H, −S-CH_2_-CH_2_-(CH_2_)_5_-C*H*_3_).^13^C NMR (75 MHz, CDCl_3_, TMS) δ 146.4 (Ar-1,3), 127.5 (Ar-2), 125.1 (Ar-5), 124.3 (Ar-4,6), 47.5 (*C*(CH_3_)_2_), 34.1 (-S-CH_2_-*C*H_2_-(CH_2_)_5_-CH_3_), 31.8 (-S-(CH_2_)_5_-*C*H_2_-CH_2_-CH_3_), 30.4 (C(*C*H_3_)_2_), 29.5 (-S-(CH_2_)_3_-*C*H_2_-(CH_2_)_3_-CH_3_), 29.2 (-S-(CH_2_)_4_-*C*H_2_-(CH_2_)_2_-CH_3_), 28.4 (-S-(CH_2_)_2_-*C*H_2_-(CH_2_)_4_-CH_3_), 24.6 (-S-*C*H_2_-(CH_2_)_6_-CH_3_), 22.6 (-S-(CH_2_)_6_-*C*H_2_-CH_3_), 14.1 (-S-(CH_2_)_7_-*C*H_3_).

Production cost analysis of **1** in comparison with commercial DOS is given in the Supplementary Information. The production cost of **1** is less than or similar to that of commercial DOS.

### Solvent extraction studies

Liquid-liquid extraction experiments were conducted in duplicate at 20±1 °C. Extraction percentages (*E*%) were calculated to ±5%. Solvent extraction studies were conducted using extractants diluted to the desired concentration in kerosene (10 mL) and the desired concentration of metal ions in acid solution (10 mL). The aqueous and organic phases were shaken thoroughly using a mechanical shaker at a speed of 300 rpm for 60 min except for studies of contact time. The aqueous and organic phases were separated with a separatory funnel. Metal ion concentrations in the aqueous phase were determined by inductively coupled plasma–atomic emission spectrometry (ICP–AES). Metal concentration and *E*% were calculated using Equations (1)–(2):

1 $$E \% ={[{\rm{M}}]}_{{\rm{org}}}/\,{[{\rm{M}}]}_{\mathrm{aq},\mathrm{init}}\times 100$$

2 $${[{\rm{M}}]}_{{\rm{org}}}=({[{\rm{M}}]}_{\mathrm{aq},\mathrm{init}}-{[{\rm{M}}]}_{{\rm{aq}}})$$

where [M]_aq,init_ is the initial metal ion concentration, and [M]_aq_ is the final metal ion concentration in aqueous solution. Other conditions are given in the Results and Discussion section. The organic-to-aqueous phase volume ratio (O/A) was constant for all extraction experiments except for studies of the effect of the O/A ratio. The concentration of commercial extractants (DOS or LIX^®^ 84-I) was twice that of synthesise﻿d extractants (**1**–**4**) except for Pd(II) loading capacity studies. In that case, the effect of HCl concentration was studied using 1 mM Pd(II) in 0.1–8.0 M HCl with 1 mM (**1**–**4**) or 2 mM (DOS) extractants diluted in kerosene for 60 min. Otherwise, the effect of HCl concentration was studied using 1 mM Pd(II) in 0.1–8.0 M HCl with 10 mM **1**–**4** or 20 mM DOS or LIX^®^ 84-I diluted in kerosene for 60 min. The Pd(II) extraction rate was determined by varying the contact time from 5–250 min using 1 mM Pd(II) in 8.0 M HCl with 10 mM **1** or **2** or 20 mM DOS. The effect of HNO_3_ concentration was studied using 1 mM Pd(II) in 0.1–8.0 M HNO_3_ with 10 mM **1**–**4** or 20 mM DOS for 60 min. The effect of mixed HCl + HNO_3_ concentrations on Pd(II) extraction was studied using 1 mM Pd(II) in combination with 0.1–6.0 M HCl and 0.25–2.0 M HNO_3_ and 10 mM **1** or **2** or 20 mM or DOS. The effect of diluents on Pd(II) extraction was carried out with 1 mM Pd(II) in 3 M HCl by diluting 10 mM **1** or **2** in high boiling-point commercial diluents. Pd(II) extractability of **1**, **2**, and DOS in HCl + HNO_3_ at various times (1–168 h) was studied using 1 mM Pd(II) in 2.0 M HCl + 1.0 M HNO_3_ solution with 10 mM **1** or **2** and 0.1 M DOS. Studies of the effect of organic-to-aqueous phase volume ratio (O/A) on Pd(II) extraction were conducted using 10 mM **1** or **2** or 20 mM DOS with 1 mM Pd(II) in 8.0 M HCl by varying O/A between 1:5 and 1:1. The effect of Pd(II) loading on extractants was studied using 10 mM **1** or **2** or 20 mM DOS in kerosene with initial Pd(II) concentration of 200–1800 mg/L in 8.0 M HCl. The experiments were carried out with O/A = 1 for 60 min. The Pd(II) loading capacity of **1**, **2﻿﻿**, or DOS was calculated according to equation (2). Pd(II) extraction was carried out from simulated solutions containing a mixture of base metals, PGMs, and rare earth metals (Pd, Pt, Rh, Y, Zr, Ba, Al, La, Ce, Cu, Fe, Zn, and Ni) that simulate the leach liquor of primary and secondary sources of PGMs. Mixed metal solutions containing 0.1 g L^−1^ each of Pd, Pt, Rh, Y, Zr, Ba, Al, La, Ce, Cu, Fe, Zn, and Ni were prepared from the corresponding chloride salts in 3.0 M HCl. 10 mM **1** or **2** or 20 mM DOS was shaken with the simulated solution for 60 min.

### Acid stability studies of 1, 2, and DOS

Approximately 1 mL of **1**, **2**, or DOS was diluted in 10 mL of CHCl_3_ and stirred with 10 mL of 12.0 M HCl or 2.0 M HCl + 1.0 M HNO_3_ for 7 days, after which the extractants were separated by evaporation of the CHCl_3_ phase and analysed by FT-IR and ^1^H NMR spectroscopy.

### Preparation of acid leach liquors of automotive catalysts and their extraction studies

Automotive catalysts procured from commercial resources in Japan served as the secondary source of PGMs. Automotive catalysts pellets were milled and pre-treated by hydrogen reduction. Samples were finely ground and sieved to less than 500 μm. A simple and effective PGMs leaching process developed by our group was adopted for the present study^43^. PGM leaching was carried out using HCl (11.6 M) and H_2_O_2_ (1 vol%) at 65 °C for 3 h. The ratio of solid to liquid (pulp density) during leaching was 500 g L^−1^. Dissolution of PGMs was 95.5, 100, and 85.6% for Pt, Pd, and Rh, respectively. The concentrations of leached metal ions (Rh, Pd, Pt, Zr, Ce, Ba, Al, La, and Y) were determined by ICP-AES. Acid-leached solutions were diluted to five times the original volume with distilled water for liquid−liquid extraction studies. The pH of the diluted PGM solution was 0.8 (~0.1 M HCl). 10 mM **1** or **2** in kerosene (10 mL) was mixed with the diluted leach liquors (10 mL) and shaken at 300 rpm for 60 min. The Pd(II) loading capacities of 10 mM **1** or **2** or 20 mM DOS in kerosene were performed by repeated contact of the leach liquors of automotive catalysts with O/A = 1 for 60 min.

### Stripping of Pd(II) and reusability of extractants 1 and 2

Stripping of Pd(II) ions from 10 mL organic phases containing 10 mM **1** or **2** and leached metal ions was performed with 1 M HCl, 1 M HNO_3_, 1 M H_2_SO_4_, 5% aqueous NH_3_, and 0.1 M thiourea + 1.0 M HCl (10 mL). The Pd(II) stripping percentage (*S*%) was calculated using the following equation:

3 $${\rm{S}}( \% )={[{\rm{Pd}}({\rm{II}})]}_{{\rm{aq}}}/{[{\rm{Pd}}({\rm{II}})]}_{{\rm{org}}}\times 100$$

where [Pd(II)]_aq_ is the Pd(II) concentration in the aqueous solution after stripping, and [Pd(II)]_org_ is the concentration in the organic phase before stripping. After stripping Pd(II), 10 mL of metal-free organic phases of **1** and **2** were washed with 20 mL of water and reused for five successive extraction/stripping cycles of automotive catalyst leach liquors under the same conditions.

## Conclusions

Pd(II) extraction behaviour of SCS pincer ligands **1**‒**4** in kerosene was studied in HCl and HNO_3_. Extractants **1** and **2** showed excellent extractability (*E*% = 99.9–98.0%) from 0.1–8.0 M HCl or HNO_3_ and from mixed HCl + HNO_3_ media. The Pd(II) *E%* of commercial DOS was 99.1‒90.2% from HCl (0.1‒8.0 M) and 99.9–72.0% from HNO_3_ (0.1–8.0 M). Extractant **3** and a commercial oxime extractant, LIX^®^ 84-I, showed poor Pd(II) extraction from HCl. Ligand **4** formed a third phase with kerosene. Extractants **1** and **2** extracted Pd(II) at a faster rate than DOS. **1** was stable in HCl and HCl + HNO_3_ solutions, whereas **2** and DOS were oxidised in HCl + HNO_3_ media. Extractant **1** was very resistant to oxidation, and its Pd(II) *E*% was unaffected in aqueous acidic solution. An O/A phase ratio of 1:1 was optimum for complete extraction of Pd(II) with **1** and **2**. The Pd(II) loading capacities of **1** and **2** were ~1.06 g L^−1^ (10 mM); that of DOS was ~0.53 g L^−1^ (5 mM). Extractants **1** and **2** extracted twice as much Pd(II) as DOS. **1** and **2** extracted >99.9% Pd(II) in various commercial diluents (ISOPAR M, ShellSol D70^®^ and kerosene) with clear phase separation, which makes them suitable for industrial applications. **1** and **2** exhibited clear selectivity for extraction of Pd over Pt, Rh, Y, Zr, Ba, Al, La, Ce, Cu, Fe, Zn, and Ni in HCl media. Extractants **1** and **2** also displayed high selectivity and Pd(II) extractability (*E*% = 99.9%) from a solution leached from automotive catalyst residue containing Pd(II), Pt(IV), Rh(III), Zr(IV), La(III), Ce(III), Y(III), Ba(II), and Al(III). Complete Pd(II) back extraction from **1** and **2** was achieved with an aqueous solution of 0.1 M thiourea and 1 M HCl. Efficiency of Pd(II) extraction and back extraction of recycled **1** and **2** was >99.9% after five extraction/stripping cycles. Extractants **1** and **3** remove Pd by forming an SCS Pd-pincer complex in which the aromatic sp^2^ C(2)–H bond is activated to enable formation of five-membered chelate rings containing a Pd–C bond. Extractants **2** and **4** do not form a pincer complex and extract Pd(II) solely via sulphur coordination. **1** is stable in HCl and HNO_3_, possesses good Pd(II) extraction capability from these media, and can be synthesized inexpensively in high yield at room temperature. **1** is suitable for recovering Pd(II) from acid leach liquors containing various oxidising agents and can be used successfully in place of DOS for selective separation of Pd(II) in PGM refineries. The present study reports our development of the novel inexpensive SCS pincer extractant **1** and an improved approach for the recovery of Pd(II) in current refining processes.

## Electronic supplementary material

Supplementary Information
