# Supplementary material for: Synthesis of pincer-type extractants for selective extraction of palladium from PGMs: An improved liquid-liquid extraction approach to current refining processes
Source: Sci Rep. 2017 Aug 18;7:8709. doi: 10.1038/s41598-017-09053-z (PMC5562712; doi:10.1038/s41598-017-09053-z)
Supplement: Supplementary file 1 — Supplementary Information [file 41598_2017_9053_MOESM1_ESM.pdf]

Supplementary Information

Synthesis of pincer-type extractants for selective extraction of palladium from PGMs: An improved liquid-liquid extraction approach to current refining processes

Muniyappan Rajiv Gandhi[1], Manabu Yamada[2], Kazutoshi Haga[3] & Atsushi Shibayama[4]

[1]Graduate School of International Resource Sciences, Akita University, Akita 010-8502, Japan.

[2]Research Center for Engineering Science, Graduate School of Engineering Science, Akita University, Akita 010-8502, Japan.

[3] Department of Applied Chemistry Course, Graduate School of Engineering Science, Akita University, Akita 010-8502, Japan.

[4]Department of Earth Resource Engineering and Environmental Science, Graduate School of International Resource Sciences Akita University, Akita 010-8502, Japan.

Correspondence and requests for materials should be addressed to M.Y. (email: myamada@gipc.akita-u.ac.jp)

Supplementary figures

Figure S1**.** Photographs of Pd(II) extraction behavior of **1**–**4** in kerosene.

Figure S2. *E%* of Pd(II) in mixed HCl-HNO_3 media in **1**, **2**, and DOS.

Figure S3. ^{1}H NMR spectra of native DOS and HCl-HNO_3 treated DOS.

Figure S4. FT-IR spectra of native **2** and 2.0 M HCl-1.0 M HNO_3 treated **2.**

Figure S5. ^{1}H NMR spectra of native **2** and 2.0 M HCl-1.0 M HNO_3 treated **2.**

Figure S6 FT-IR spectra of native **1** and 2.0 M HCl-1.0 M HNO_3 treated **1.**

Figure S7. ^{1}H NMR spectra of native **1** and 2.0 M HCl-1.0 M HNO_3 treated **1.**

Figure S8. The effect of O/A ratio on Pd(II) extractability of **1**, **2**, and DOS.

Figure S9. Pd(II) loading capacities of **1**, **2**, and DOS in kerosene.

Figure S10. Pd(II) loading capacities of **1**, **2**, and DOS in kerosene from diluted automotive catalysts leach liquors.

Figure S11. Color changes during stripping of Pd(II) from organic phase of **1** and **2** using acidic thiourea solution.

Figure S12. FT-IR spectra of **1**, **2** and their **1**-Pd, **2**-Pd complexes.

Figure S13. FT-IR spectra of **3**, **4** and their **3**-Pd, **4**-Pd complexes.

Figure S14. UV-visible spectra of **1**–**4**, Pd(II) solution and their extractant- Pd complexes.

Figure S15. Carbon numbers in the aromatic ring for extractant **1**–**4**.

Figure S16. ^{1}H NMR spectra of **1** and **1**-Pd complex.

Figure S17. ^{1}H NMR spectra of **2** and **2**-Pd complex.

Figure S18.^{1}H NMR spectra of **3** and **3**-Pd complex.

Figure S19. ^{1}H NMR spectra of **4** and **4**-Pd complex.

Figure S20. Synthesis of extractant **1.**

Supplementary Tables

Table S1. The results of ^{1}H NMR analysis of **1** and **1**-Pd complex.

Table S2. The results of ^{1}H NMR analysis of **2** and **2**-Pd complex.

Table S3. The results of ^{1}H NMR analysis of **3** and **3**-Pd complex.

Table S4. The results of ^{1}H NMR analysis of **4** and **4**-Pd complex.

Supplementary details

Production cost analysis of **1** in comparison with commercial DOS (Di-*n*-octyl Sulfide).

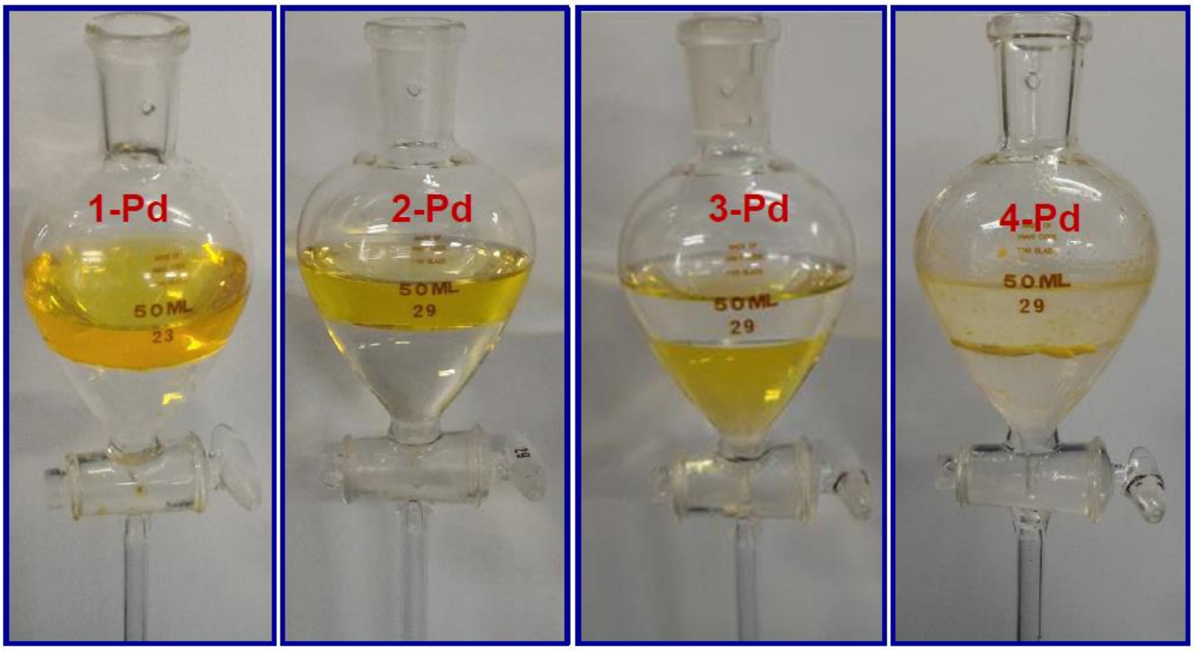

Figure S1. Photographs of Pd(II) extraction behavior of **1**–**4** in kerosene.
[The experimental conditions are those referred in Fig. 2 with 0.1 M HCl solution]

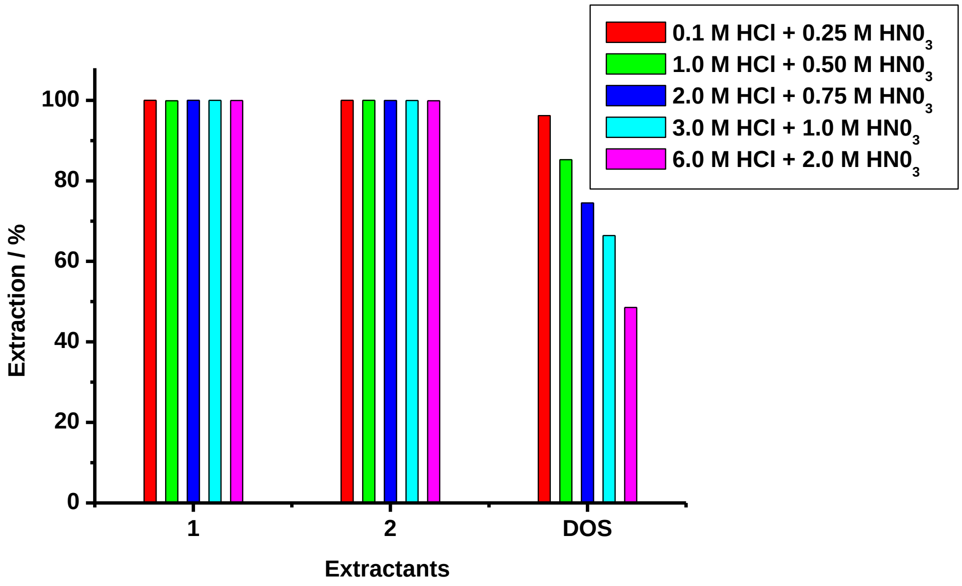

Figure S2. *E*% of Pd(II) in mixed HCl-HNO_3 media in **1**, **2**, and DOS.

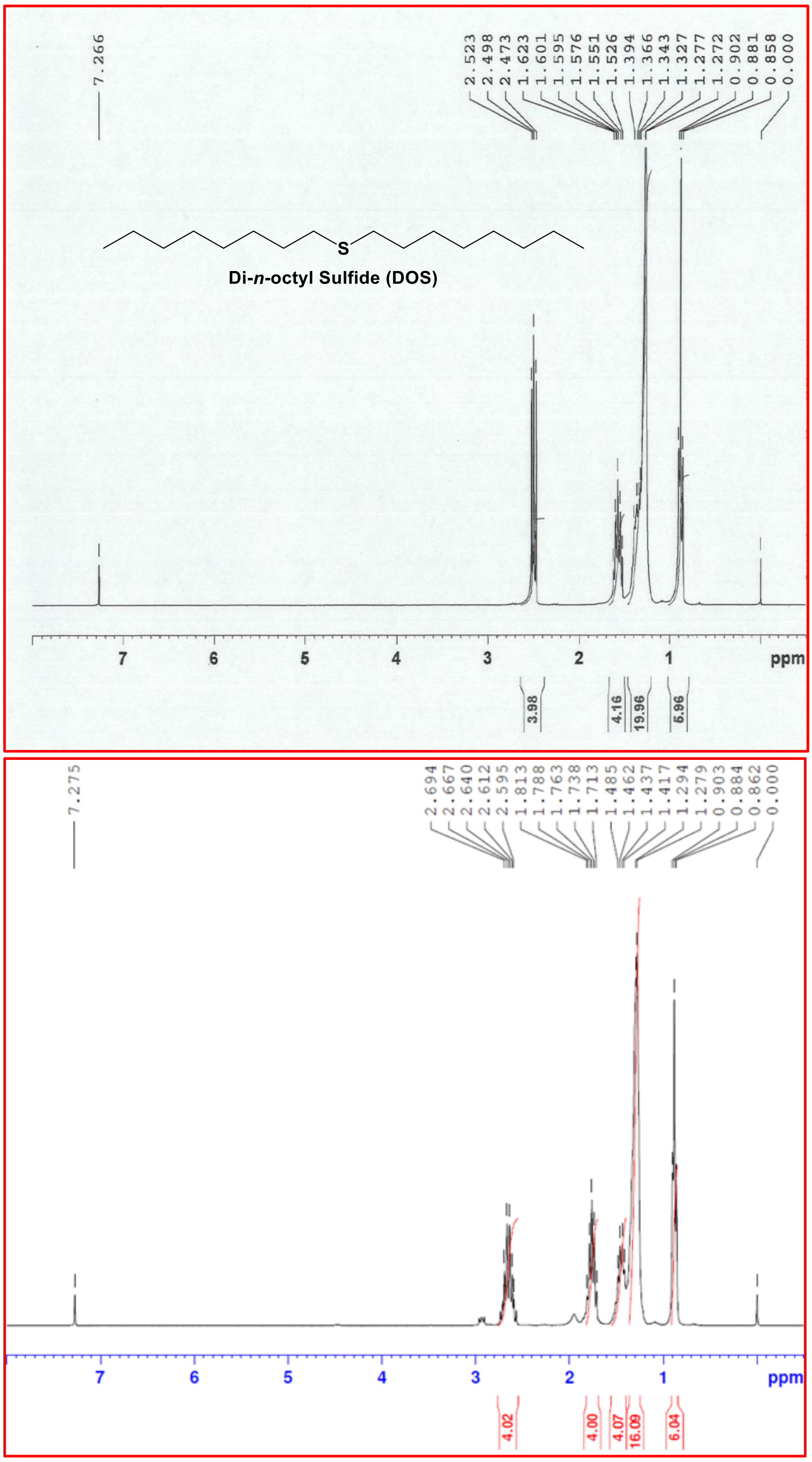

Figure S3. ^{1}H NMR spectra of native DOS and HCl-HNO_3 treated DOS.

<u>^{1}H NMR peaks of DOSO reported in Reference 42 is given below:</u>

DOSO[42] : ^{1}H-NMR ($CDCl_3$) δ 0.85 (t, 6H, $2CH_3$), 1.24 (m, 16H, $8CH_2$), 1.41 (m, 4H, $2CH_2$) 1.73 (m, 4H, $8CH_2$) 2.61 (m, 4H, $2CH_2S$).

^{1}H NMR spectra of HCl-HNO_3 treated DOS is exactly matching with ^{1}H NMR spectra of DOSO reported in reference 42 which confirms that DOS is oxidised to DOSO by HCl-HNO_3 treatment.

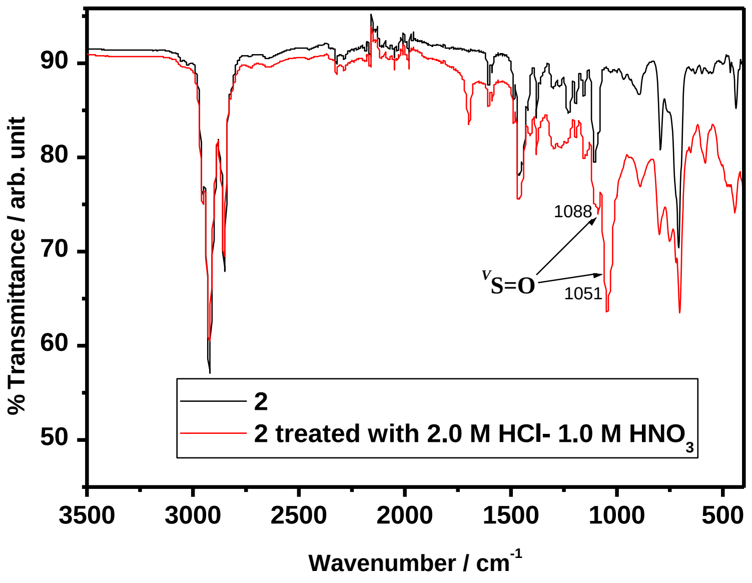

Figure S4. FT-IR spectra of native **2** and 2.0 M HCl-1.0 M HNO_3 treated **2.**

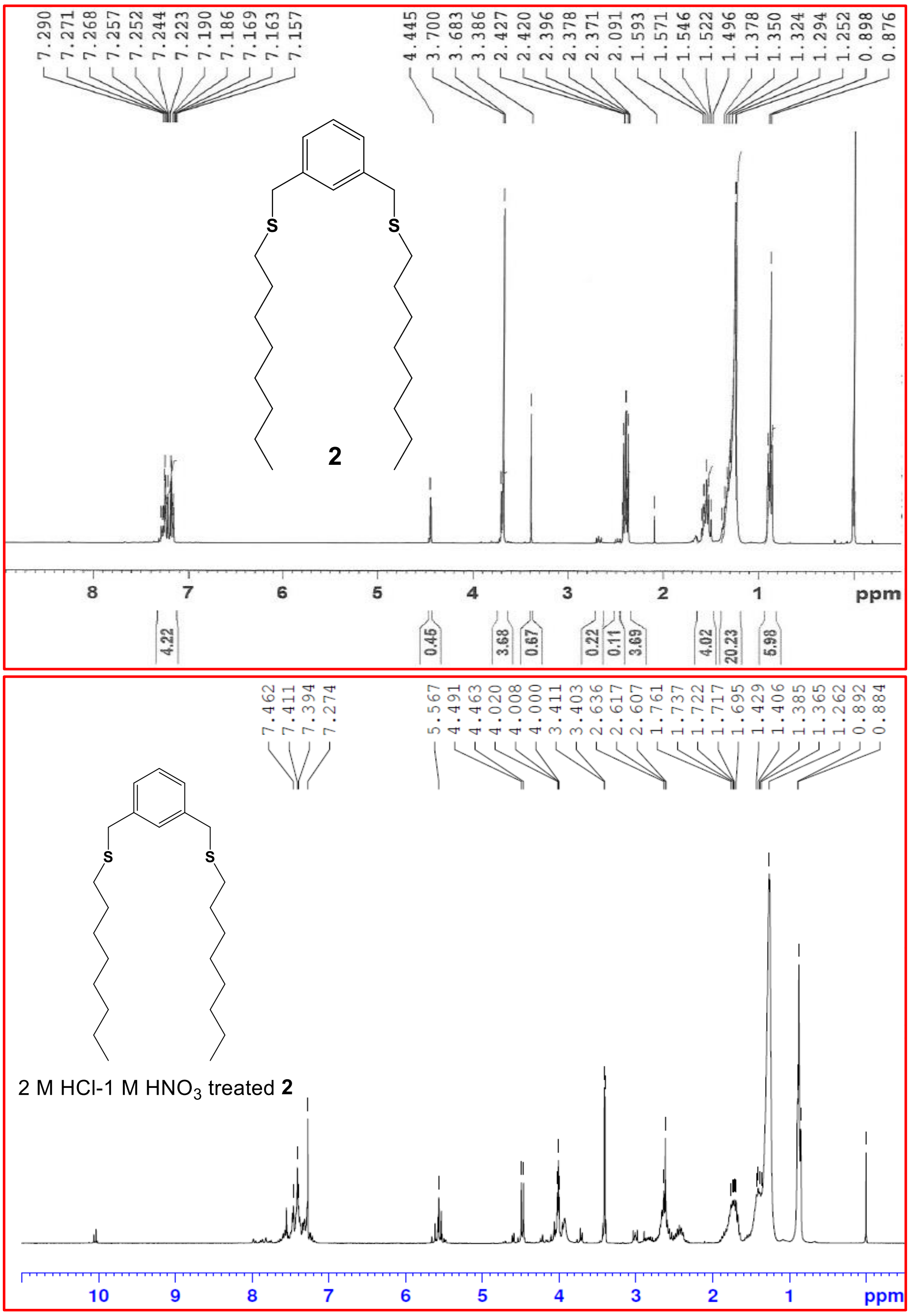

Figure S5. ^{1}H NMR spectra of native **2** and 2.0 M HCl-1.0 M HNO_3 treated **2**.

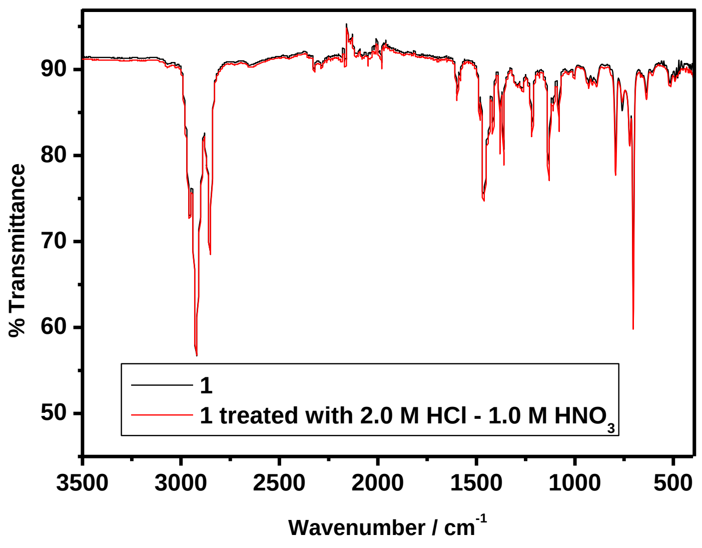

Figure S6. FT-IR spectra of native **1** and 2.0 M HCl-1.0 M HNO_3 treated **1**.

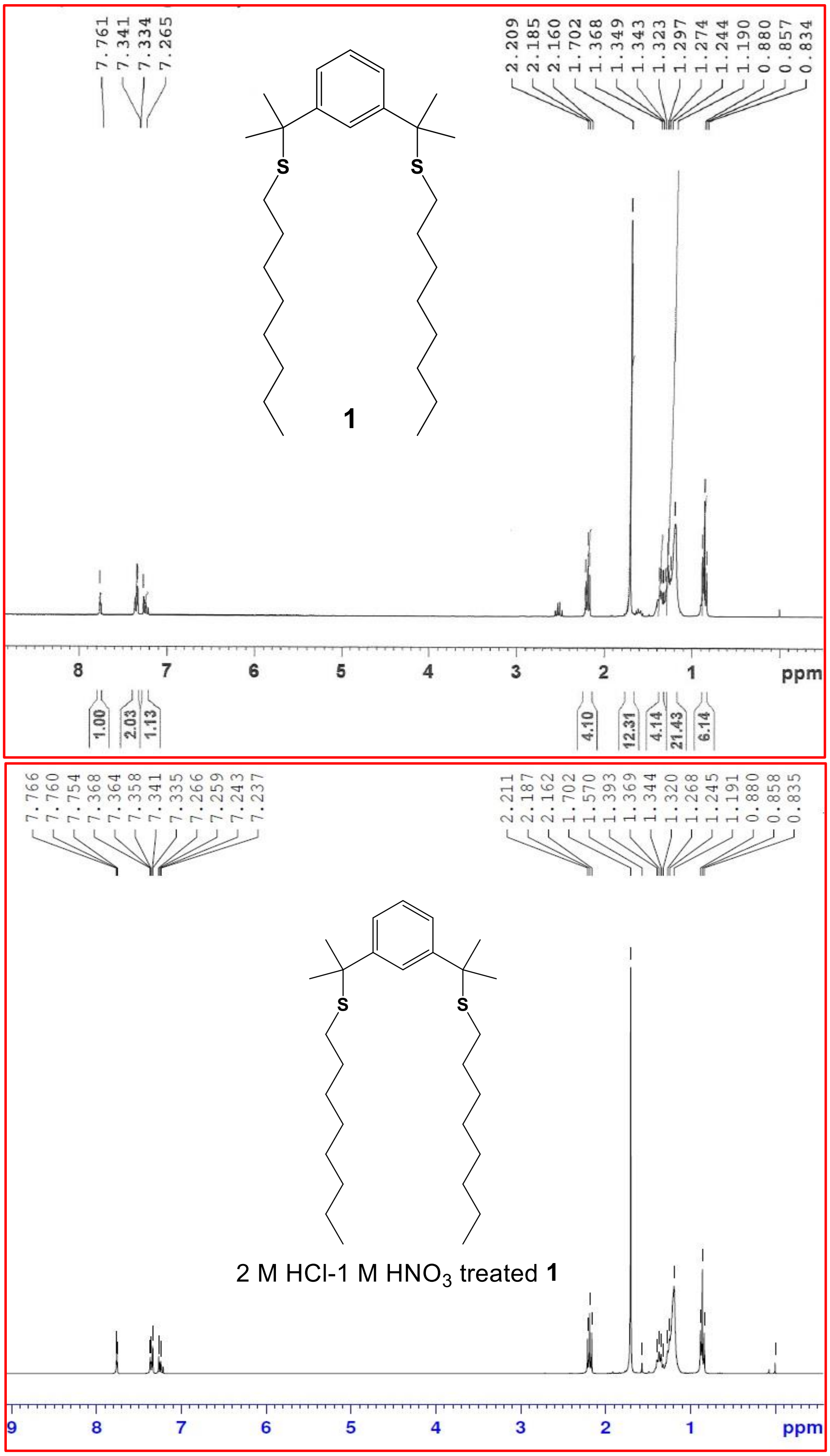

Figure S7. ^{1}H NMR spectra of native **1** and 2.0 M HCl-1.0 M HNO_3 treated **1**.

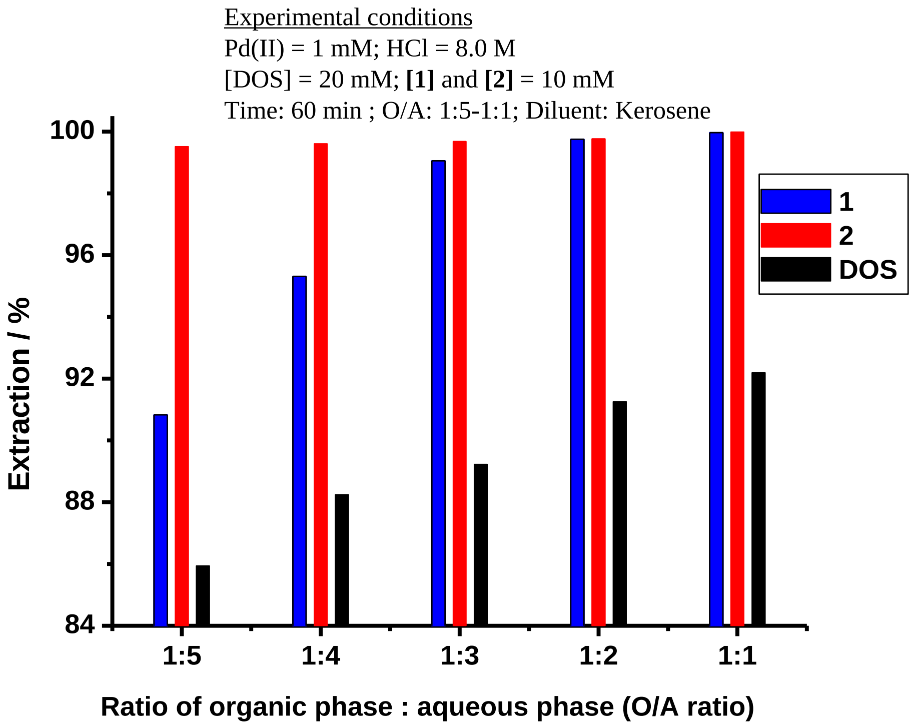

Figure S8. The effect of O/A ratio on Pd(II) extractability of **1**, **2**, and DOS.

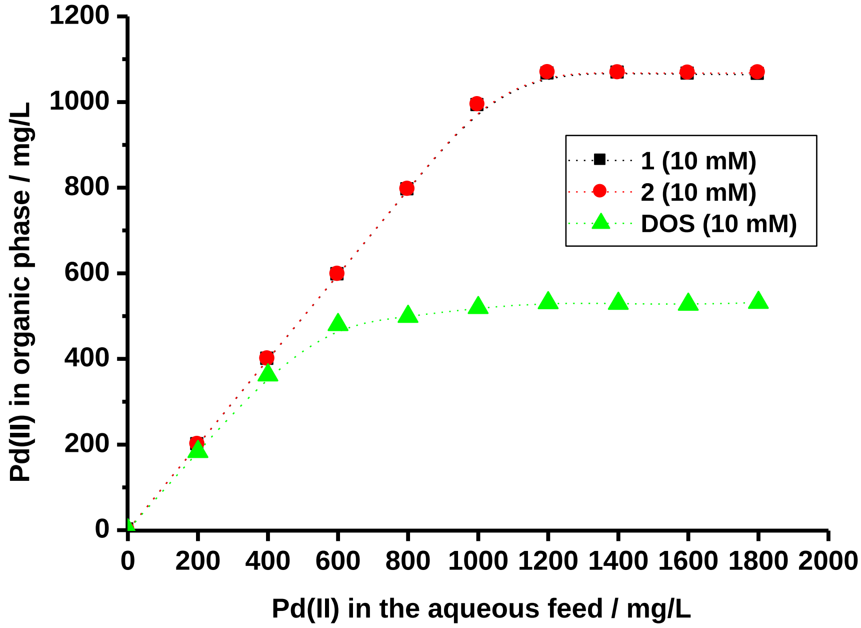

Figure S9. Pd(II) loading capacities of **1**, **2**, and DOS in kerosene.

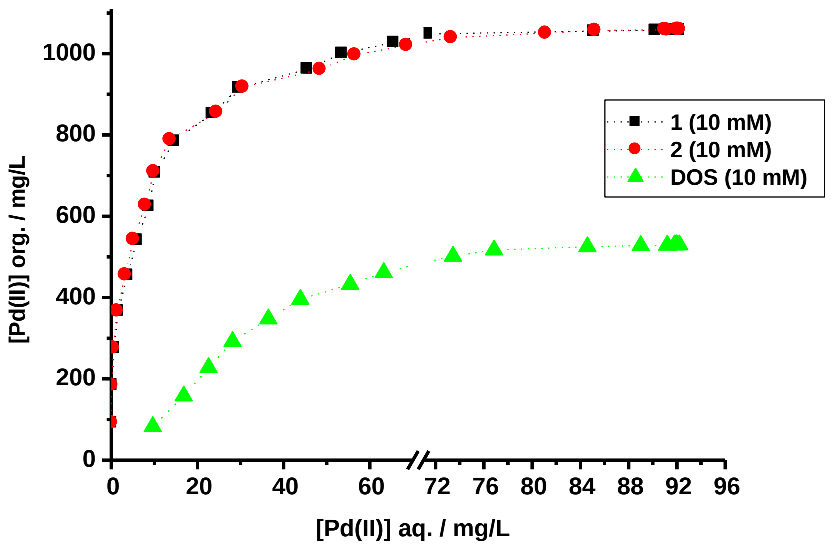

Figure S10. Pd(II) loading capacities of **1**, **2**, and DOS in kerosene from automotive catalysts leach liquors.

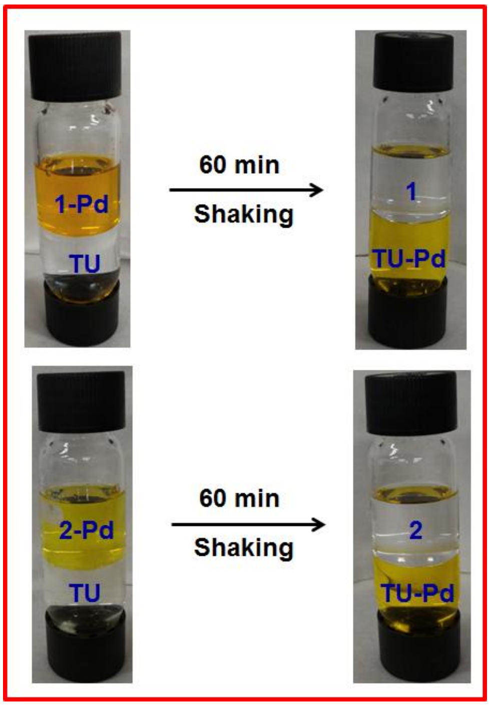

Figure S11. Color changes during stripping of Pd(II) from organic phase of **1** and **2** using acidic thiourea solution.

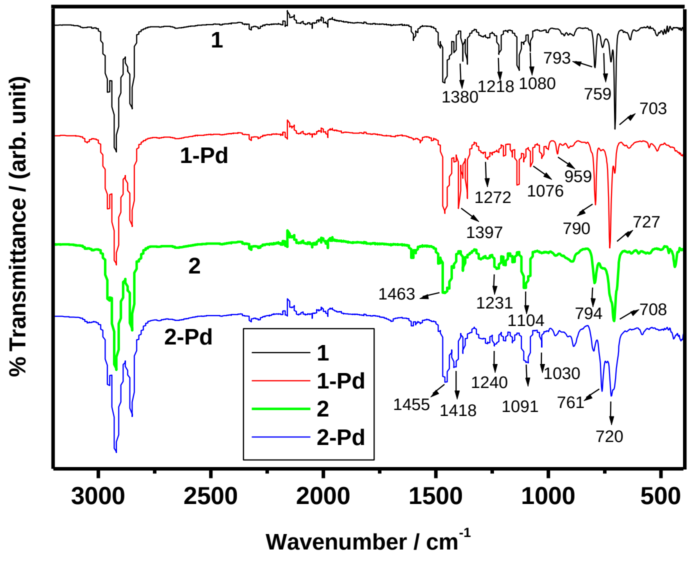

Figure S12. FT-IR spectra of **1**, **2** and their **1**-Pd, **2**-Pd complexes.

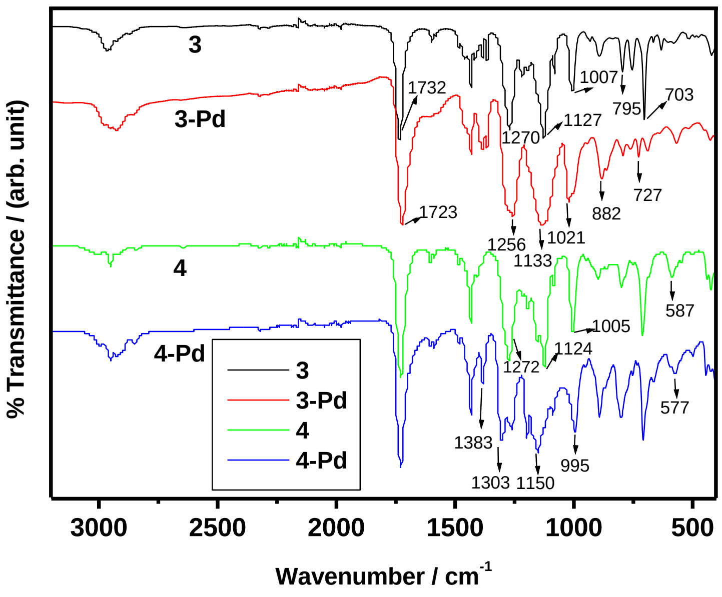

Figure S13. FT-IR spectra of **3**, **4** and their **3**-Pd, **4**-Pd complexes.

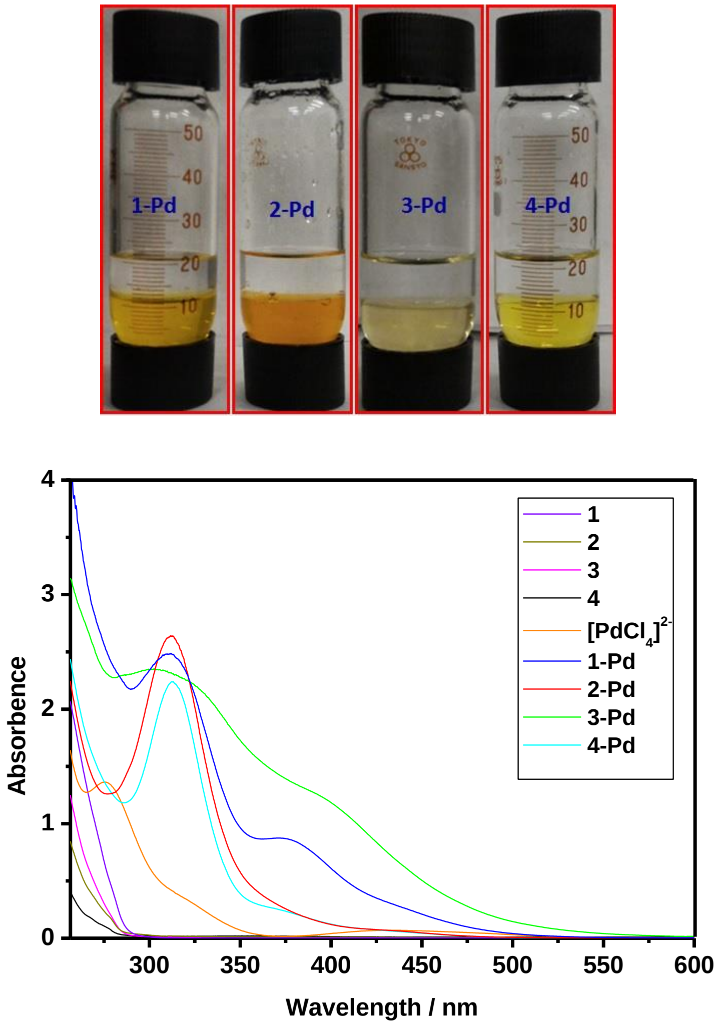

Figure S14. UV-visible spectra of **1-4**, Pd(II) solution and their extractant-Pd complexes.

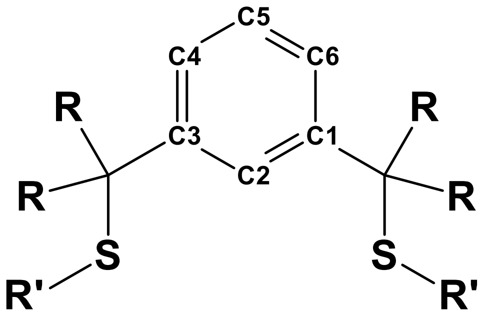

1: R = CH_3; R'= -$CH_2(CH_2)_6CH_3$

2: R= H; R'= -$CH_2(CH_2)_6CH_3$

3: R = CH_3; R'= -CH_2COOMe

4: R = H; R'= -CH_2COOMe

Figure S15. Carbon numbers in the aromatic ring for extractants **1-4**.

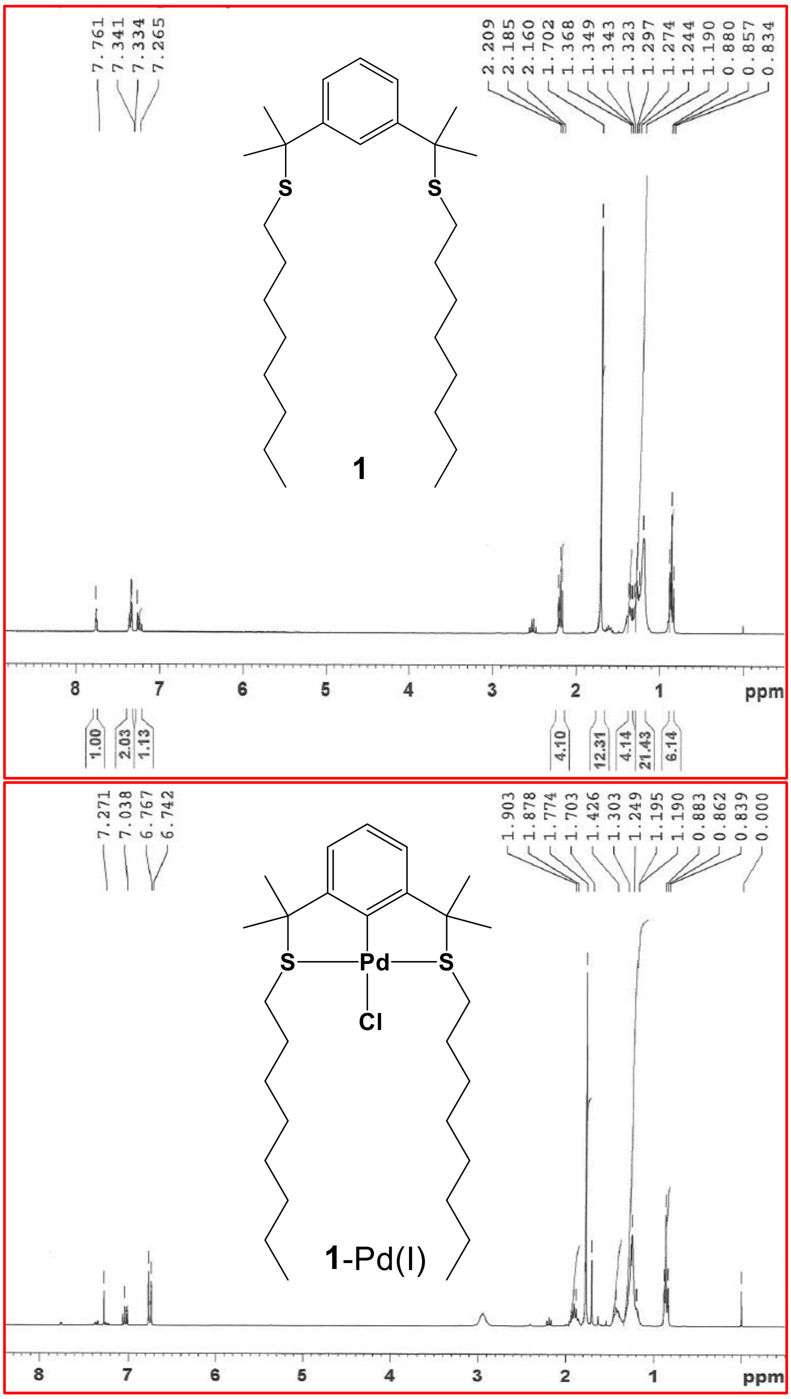

Figure S16. ^{1}H NMR spectra of **1** and **1**-Pd complex.

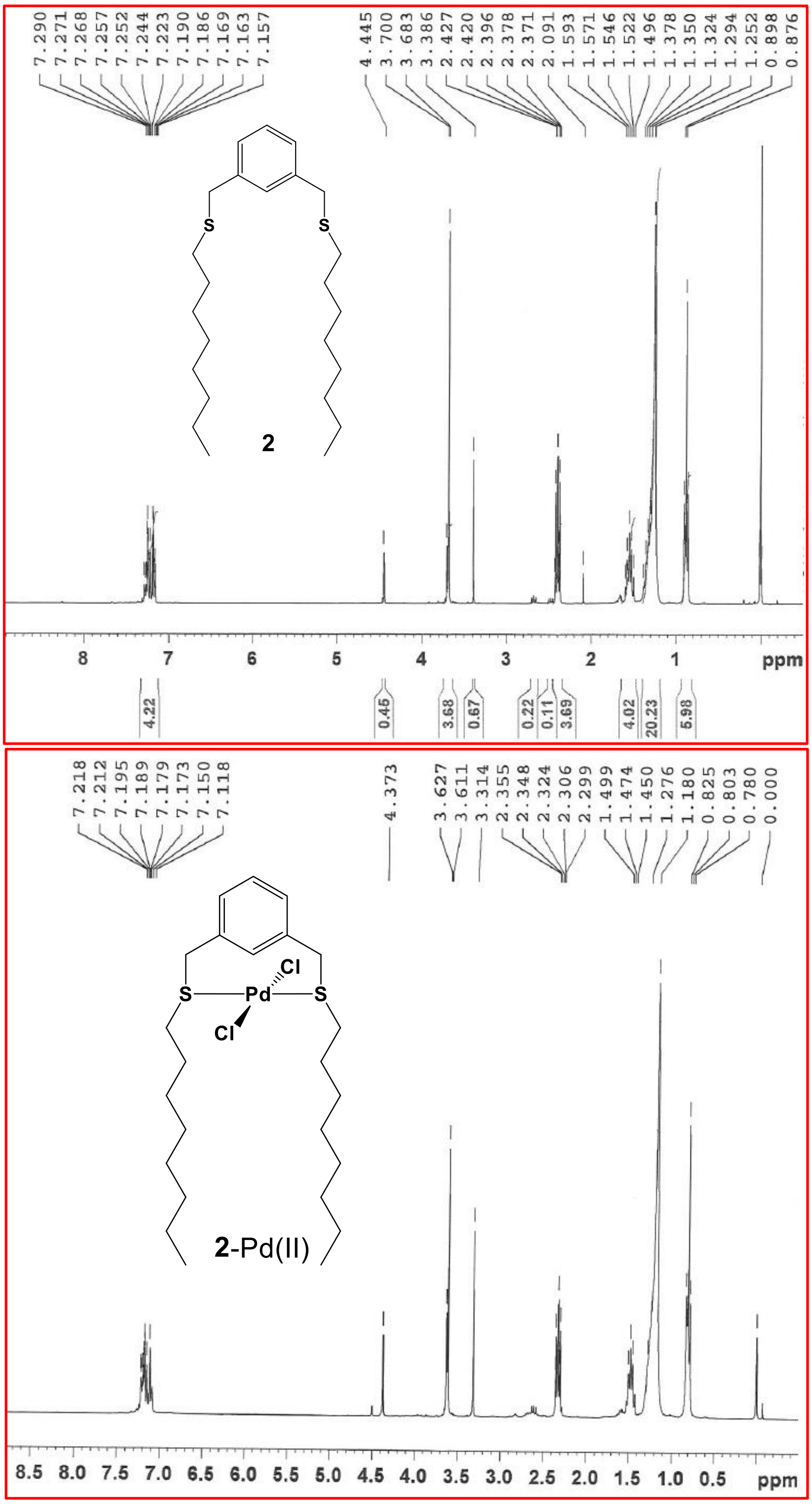

Figure S17. ^{1}H NMR spectra of **2** and **2**-Pd complex.

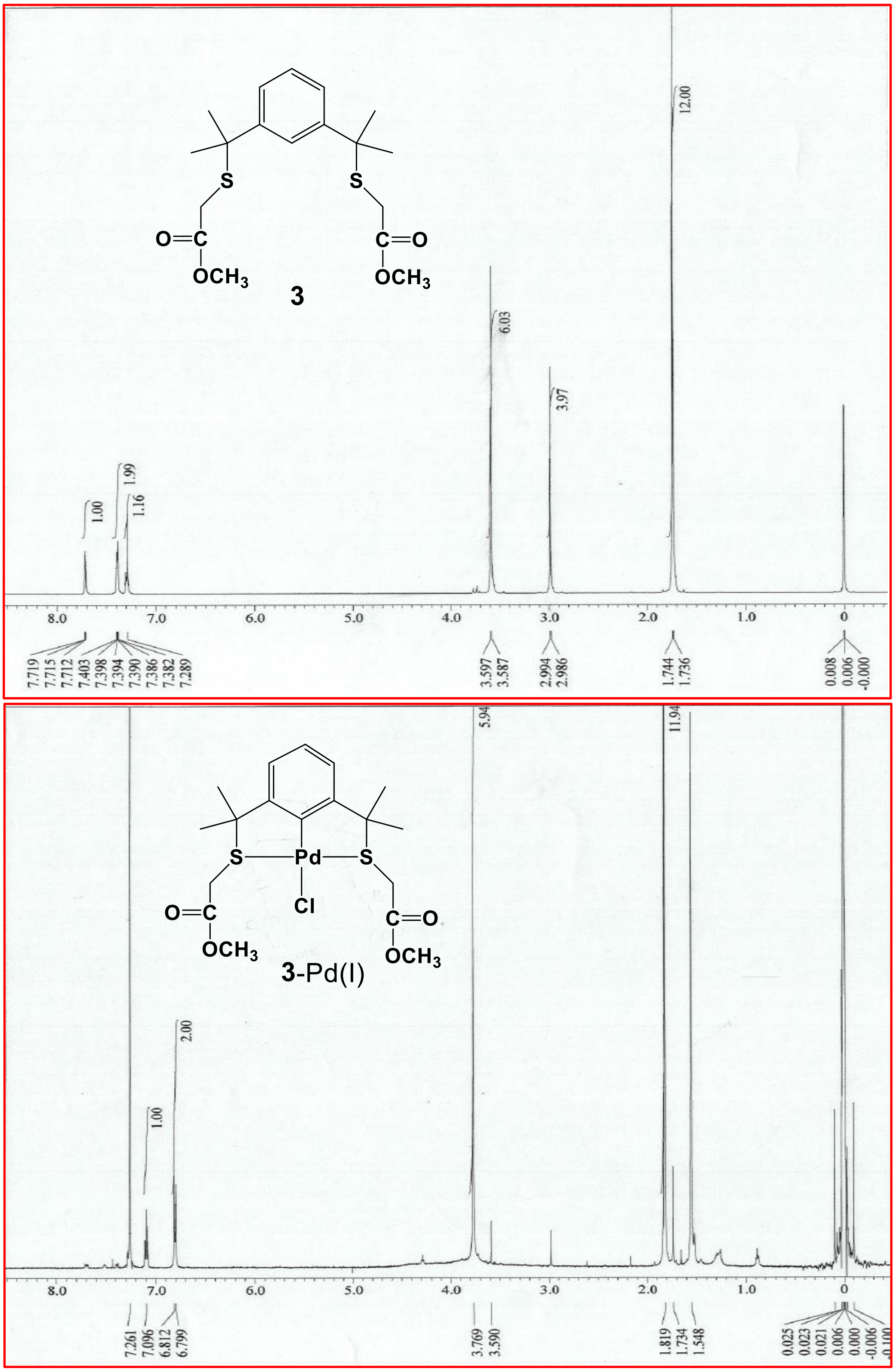

Figure S18.^{1}H NMR spectra of **3** and **3**-Pd complex.

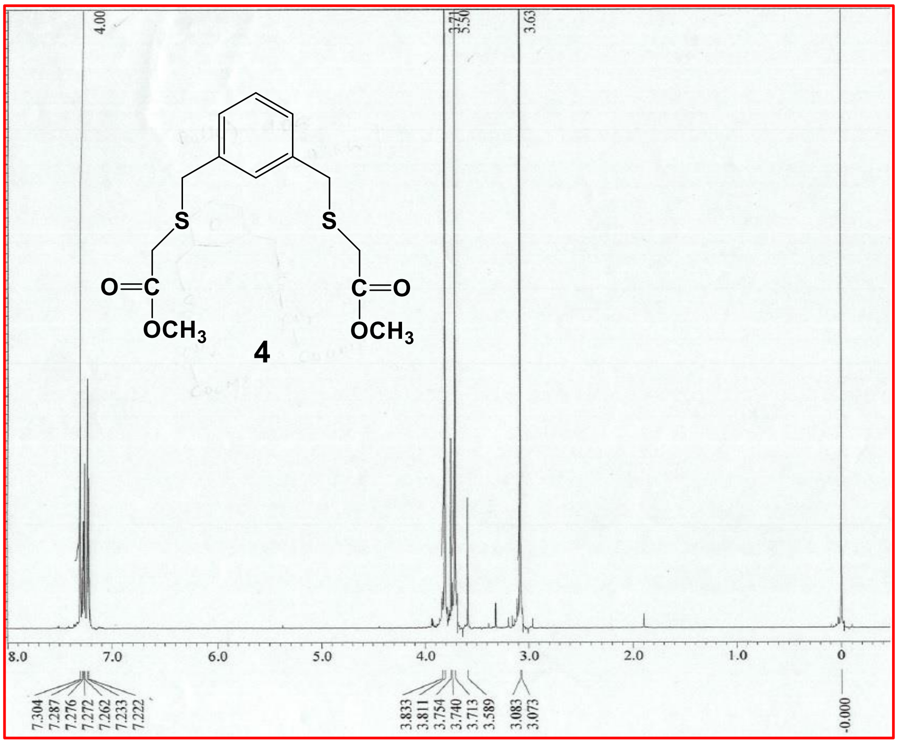

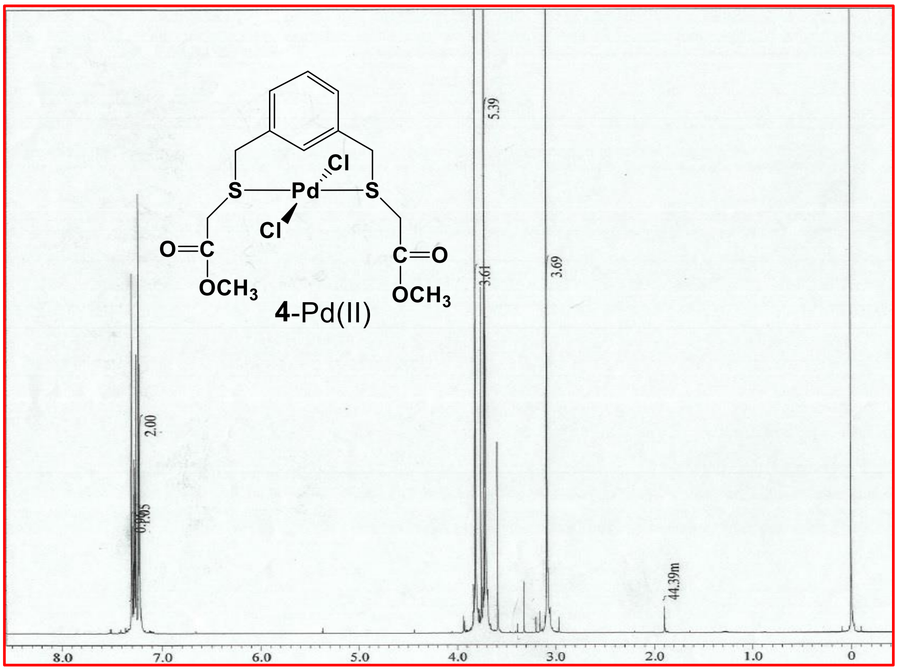

Figure S19. ^{1}H NMR spectra of **4** and **4**-Pd complex.

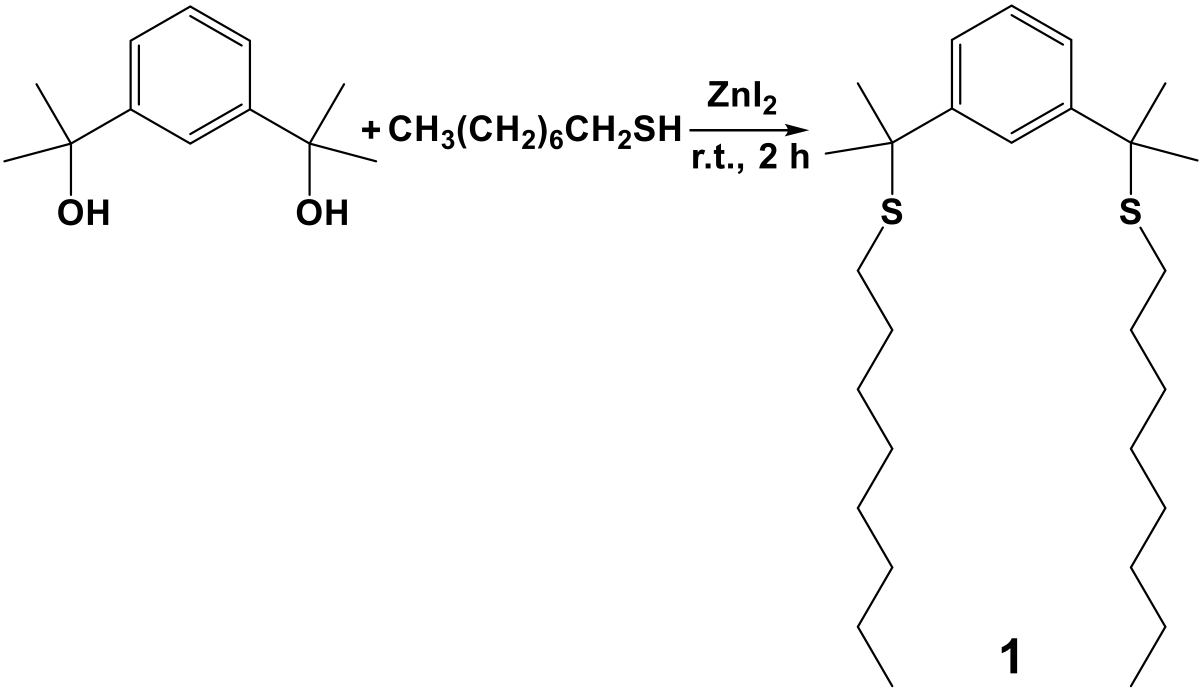

Figure S20. Synthesis of extractant **1.**

Table S1. The results of ^{1}H NMR analysis of **1** and **1**-Pd complex.

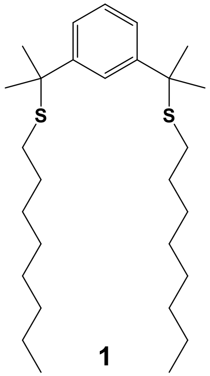 **1**	7.76 (t, 1H, Ar-C2-*H*)[a]	7.33 (dd, 2H, Ar-C4,6-*H*)[a]	7.26 (t, 1H, Ar-C5-*H*)[a]	2.18 (t, 4H, -S-CH_2-CH_2-(CH_2)$_5$-CH_3)	1.70 (s, 12H, Ar-(CH_3)$_2$-S-)	1.34 (m, 4H, -S-CH_2-CH_2-(CH_2)$_5$-CH_3),	1.32-1.19 (m, 20H, -S-CH_2-CH_2-(CH_2)$_5$-CH_3)	0.85 (t, 6H, -S-CH_2-CH_2-(CH_2)$_5$-CH_3).
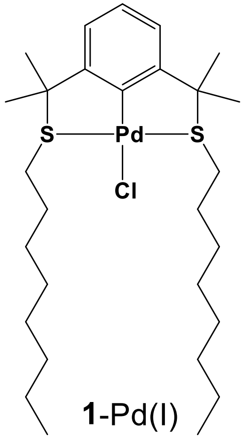 **1**-Pd(I)	-	6.74 (dd, 2H, Ar-C4,6-*H*)[a]	7.03 (t, 1H, Ar-C5-*H*)[a]	1.87 (m, 4H, -S-CH_2-CH_2-(CH_2)$_5$-CH_3)	1.77 (s, 12H, Ar-(CH_3)$_2$-S)	1.30 (m, 4H, -S-CH_2-CH_2-(CH_2)$_5$-CH_3)	1.24-1.19 (m, 20H, -S-CH_2-CH_2-(CH_2)$_5$-CH_3),	0.86 (t, 6H, -S-CH_2-CH_2-(CH_2)$_5$-CH_3).

[a] C2, C4, C5 and C6 show carbon number in each aromatic ring.

Table S2. The results of ^{1}H NMR analysis of **2** and **2**-Pd complex.

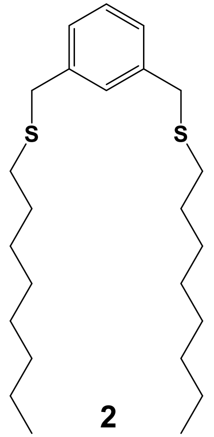 **2**	7.29-7.15 (m, 4H, Ar)	3.68 (s, 4H, Ar-CH_2-S-)	2.39 (t, 4H, -S-CH_2-CH$_2$-(CH$_2$)$_5$-CH$_3$)	1.52 (m, 4H, -S-CH$_2$-CH_2-(CH$_2$)$_5$-CH$_3$)	1.32 (m, 20H, -S-CH$_2$-CH$_2$-(CH_2)$_5$-CH$_3$),	0.89 (t, 6H, -S-CH$_2$-CH$_2$-(CH$_2$)$_5$-CH_3).
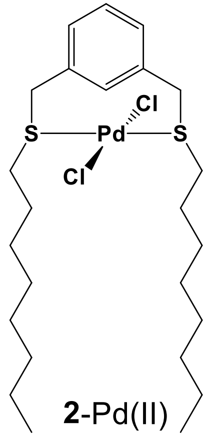 **2**-Pd(II)	7.21-7.11 (m, 4H, Ar)	3.61 (s, 4H, Ar-CH_2-S-)	2.30 (t, 4H, -S-CH_2-CH$_2$-(CH$_2$)$_5$-CH$_3$)	1.47 (m, 4H, -S-CH$_2$-CH_2-(CH$_2$)$_5$-CH$_3$)	1.18 (m, 20H, -S-CH$_2$-CH$_2$-(CH_2)$_5$-CH$_3$),	0.80 (t, 6H, -S-CH$_2$-CH$_2$-(CH$_2$)$_5$-CH_3).

Table S3. The results of ^{1}H NMR analysis of **3** and **3**-Pd complex.

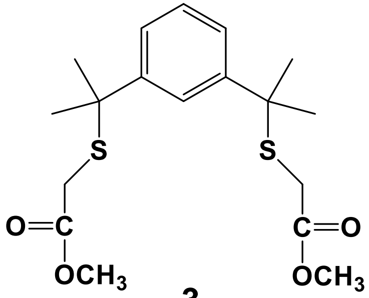 **3**	7.71 (t, 1H, Ar-C2-*H*)[a]	7.39 (dd, 2H, Ar-C4,6-*H*)[a]	7.28 (t, 1H, Ar-C5-*H*)[a]	3.58 (s, 6H, $-CO_2CH_3$)	2.98 (s, 4H, $-S-CH_{2-}$ CO_2CH_3)	1.73 (s, 12H, Ar-C$H_3)_2$-S-).
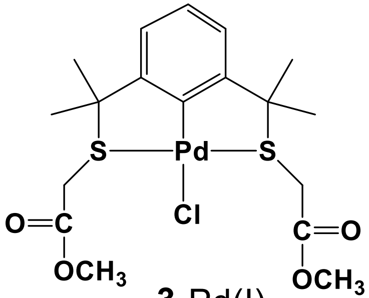 **3**-Pd(I)	-	6.79 (d, 2H, Ar-C4,6-*H*)[a]	7.09 (t, 1H, Ar-C5-*H*)[a]	3.79 (s, 6H, $-CO_2CH_3$)	4.80-3.74 (br, 4H, $-S-CH_{2-}$ CO_2CH_3)	1.81 (s, 12H, Ar-C$H_3)_2$-S-)

[a] C2, C4, C5 and C6 show carbon number in each aromatic ring.

Table S4. The results of ^{1}H NMR analysis of **4** and **4**-Pd complex.

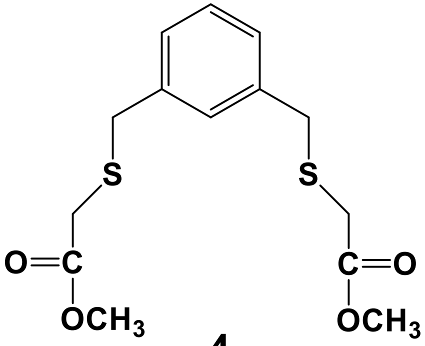 **4**	7.30–7.22 (m, 4H, Ar)	3.81 (s, 4H, Ar-CH_2-S-)	3.71 (s, 6H, -CO_2CH_3)	3.08 (s, 4H, -S-CH_2-CO_2CH_3)
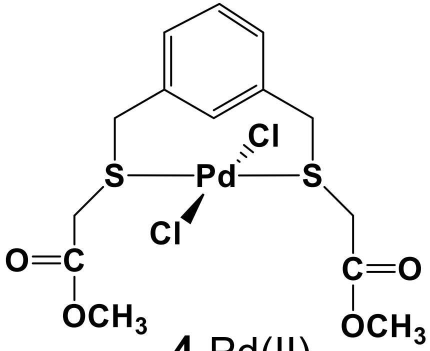 **4**-Pd(II)	7.33 −7.24 (m, 4H, Ar)	3.84 (s, 4H, Ar-CH_2-S-)	3.73 (s, 6H, -CO_2CH_3)	3.04 (s, 4H, -S-CH_2-CO_2CH_3)

Production cost analysis of 1 in comparison with commercial DOS (Di-*n*-octyl Sulfide):

Currently, the cost of 10 g of the DOS (CAS Number：2690-08-6, Product Number：O010) is **¥ 2800** at Tokyo Chemical Industry Co., Ltd. The approximate production cost for 1,3-bis(2-(octylthio)propan-2-yl)benzene (**1**) is calculated based on the our laboratory experiment and chemicals purchased from commercial sources is given below:

S No.	Starting materials	Company	Price
1	α,α'-dihydroxy-1,3-diisopropylbenzene<br>$C_{12}H_{18}O_2$= M.Wt. 194.27<br>(CAS Number：1999-85-5<br>Product Number：D1788)	Tokyo Chemical Industry Co., Ltd. Japan	25g = ¥ 2500
2	1-Octanethiol<br>$C_8H_{18}S$ = M.Wt. 146.29<br>(CAS Number：111-88-6<br>Product Number：O0025)	Tokyo Chemical Industry Co., Ltd. Japan	25 mL (21.2 g)<br>= ¥ 2200
3	ZnI_2<br>CAS Number：10139-47-6<br>M.Wt. = 319.22	Kanto Chemical Co., INC, Japan	25 g = ¥ 2900
4	1,2-dichloroethane	Kanto Chemical Co., INC, Japan	500 mL = ¥ 1400
5	1,2-dichloromethane	Kanto Chemical Co., INC, Japan	500 mL = ¥ 1200

In order to prepare 10 g of 1,3-bis(2-(octylthio)propan-2-yl)benzene (**1**) at laboratory, we require about 4.622 g (24 mole) of α,*α'*-dihydroxy-1,3-diisopropylbenzene, 7.021 g (48 mole) of 1-Octanethiol, 7.661 g (24 mmole) of ZnI_2 and about 100 mL of each 1,2-dichloroethane/1,2-dichloromethane.

After reaction, the organic phase was washed with 1 M NaOH (100 mL; 4 g) and Na_2SO_4 (5 g) to remove the impurities.

<u>The cost of production for 10 g of 1</u> (28th February, 2017)

Starting material cost

The cost of 4.622 g - α,*α'*-dihydroxy-1,3-diisopropylbenzene = ¥ 462.2

The cost of 7.021 g - 1-Octanethiol = ¥ 728.5

The cost of 7.661 g of ZnI_2 = ¥ 888.6

Solvent cost

The cost of 100 mL of 1,2-dichloroethane = ¥ 280

The cost of 100 mL of 1,2-dichloromethane = ¥ 240

Work up cost

The cost of 4 g of NaOH = ¥ 11

The cost of 10 g of Na_2SO_4= ¥ 24

[Production cost = ¥ 2079.3 (stating material) + ~ ¥ 520 solvent cost + ~¥ 35 (work up cost)]

==

Total production cost = ¥2634.3 for 10 g of **1**

Overall cost for production for 10 g of 1 is ~ ¥ 2634.3 ($ 23.4 USD) as on 28th Feb, 2017

Whereas 10 g of DOS cost sold is ¥ 2800 ($ 24.8 USD) as on 28th Feb, 2017
